# Enhancement of color and photovoltaic performance of semi-transparent organic solar cell via fine-tuned 1D photonic crystal

**DOI:** 10.1038/s41598-022-24113-9

**Published:** 2022-11-12

**Authors:** Çağlar Çetinkaya, Erman Çokduygulular, Barış Kınacı, Serkan Emik, Nihan Akın Sönmez, Süleyman Özçelik

**Affiliations:** 1 Physics Department, Faculty of Science, Istanbul University, 34134 Istanbul, Turkey; 2 Department of Engineering Sciences, Faculty of Engineering, Istanbul University-Cerrahpaşa, 34320 Istanbul, Turkey; 3 Department of Photonics, Faculty of Applied Sciences, Gazi University, 06500 Ankara, Turkey; 4 Photonics Application and Research Center, Gazi University, 06500 Ankara, Turkey; 5 Department of Chemical Engineering, Faculty of Engineering, Istanbul University-Cerrahpaşa, 34320 Istanbul, Turkey

**Keywords:** Solar cells, Solar energy and photovoltaic technology, Photonic crystals

## Abstract

Semi-transparent organic solar cells’ (ST-OSCs) photovoltaic and high optical performance parameters are evaluated in innovative applications such as power-generating windows for buildings, automobiles, and aesthetic designs in architectural and industrial products. These parameters require the precision design of structures that optimize optical properties in the visible region and aim to achieve the required photon harvest in UV and IR. These designs can be realized by integrating wavelength-selective photonics-based systems into ST-OSC to increase localized absorption in wavelengths greater than 600 nm and NIR and provide modifiable optical properties. In this study, methodologically, we followed highly detailed light management engineering and transfer matrix method-based theoretical and experimental approaches. We discussed the optimal structures by evaluating color, color rendering index, correlated color temperature, and photovoltaic performances for ST-OSCs, including one-dimensional photonic crystal (1D-PC) designed at different resonance wavelengths (λ_B_) and periods. Finally, by integrating fine-tuned (MgF_2_/MoO_3_)^N^ 1D-PC, we report the inherently dark purple-red color of the P3HT:PCBM bulk-heterojunction-based ST-OSC neutralizes with the optimal state was 0.3248 and 0.3733 by adjusting close to the Planckian locus. We also enhanced short current density from 5.77 mA/cm^2^ to 6.12 mA/cm^2^ and PCE were increased by 7.34% from 1.77% to 1.90% designed for the N = 4 period and λ_B_ = 700 nm.

## Introduction

The features of organic semiconductor-based photovoltaic (PV) technology, such as lightness, thinness and flexibility, cost-effective production, sustainability, and simple fabrication processes, make them stand out among other photovoltaic technologies both in academic studies and in the industry^1–5^. In particular, the efficiency of organic solar cells (OSC), reaching over 18% in the past few years, has increased the application potential of many new PV systems, which are designed^1,2,6–10^. At the forefront of these new photovoltaic systems are colored, transparent and semi-transparent OSCs (ST-OSC), which present the most striking properties of organic materials^1,3,11^. Recently, researchers have especially focused on their potential applications in flexible or semi-transparent solar cells due to the flexible nature of their active layers and the strong absorption coefficient of organic photoactive materials rather than aiming for high cell efficiency^1,6,12,13^.

Usually, the active layer of the solar cell is designed to absorb light outside the visible range (380–780 nm) (VR) while maintaining high transparency in VR for ST requirements^14^. However, ST requirements can be achieved by adding different photonic and material systems to the structure without changing the structure of the organic material forming the active region^1^. In addition, developing high-conductivity transparent contact systems to reduce absorption and reflection in visible light without disturbing the selective transport and band arrangement is a critical parameter in designing high-performance ST-OSCs^13^. In OSCs, opaque metal electrodes such as Ag, Au, and Al, generally thick and highly reflective in VR, are used as contact material^1,15–17^. To achieve the aim of semi-transparency in the ST-OSC design, a thin metal material is sandwiched between two anti-reflective dielectrics as the top contact, and a dielectric/metal/dielectric (DMD) structure is formed^1,15^. DMD designs offer high transparency and conductivity, low turbidity, excellent flexibility, easy fabrication, and excellent compatibility with different substrates^1,15,17^. Because transparent electrodes have inherently low reflectivity, the photon absorption of the device must be carefully adjusted to allow sufficient light to pass through the device^18^. In addition, when evaluated electrically, while the sheet resistance (R_sh_) for ITO is about 15 Ωsq^−1^, R_sh_ below 5.75 Ωsq^−1^ can be obtained in Ag-based DMD systems^19,20^.

An average visible transmittance (AVT) of 25% is an acceptable standard for window treatments^1,21^, although the transparency required for colorless ST-OSCs and window treatments depends on the operating environment. In addition, ST-OSCs can be made suitable for application in aesthetic and decorative products by providing ST colors by adjusting the wavelength ranges where the transmittance is high. However, these modifications made in the structure to change the optical characteristics of the organic material forming the active region may negatively affect the cell output parameters by changing the material's electrical performance. Therefore, light management engineering-based ST-OSC approaches have been carried out to adjust the optical properties by modifying the propagation of the electromagnetic wave in ST-OSC without changing the active organic material^1,12,22–24^. Among these approaches, the deposition of a periodic one-dimension (1D) dielectric mirror (DM) layer with photonic crystal (PC) properties on the transparent top electrode is an effective approach for improved and modifiable optical properties^12,25,26^.

1D-PCs are structures in which the dielectric constant changes periodically in only one direction, and they consist of structures with two or more dielectric constants deposited in a single direction^27,28^. Therefore, in 1D-PCs, a photonic band gap (PBG) is formed in the wavelength range where optical reflection will occur for the propagation of photons in only one direction. 1D-PCs are simple structures to fabricate as well as are low in cost^29^. In addition, it is more usual that they can be produced with the desired optical properties^27,30^. The width and reflection intensity of the PBG can be easily adjusted thanks to the thickness optimization of 1D-PC. In this way, with the integration of 1D-PCs into ST-OSCs, the absorption and transmittance characteristics can be modified without changing the properties of the active layer, mainly by adjusting the reflection characteristic. Therefore, this allows the optical properties of ST-OSCs, such as AVT, color, and color rendering index (CRI), which are determined by optical spectra, to be adjusted at desired values^31^. In addition, PCs can also be used to increase device performance by allowing internal reflection to be controlled inside the ST-OSC^12,32,33^. The first strategy for this is to integrate the PC to ST-OSC and PBG should be in the wavelength range for which the absorption spectrum of the organic material is responsible. In this way, the photocurrent density in the structure can be increased by sending the photons that are not absorbed in the active region back to the active region.

A few critical issues must be carefully considered for ST-OSC designs with high optical performance within the optical limits of the organic material forming the active region: (1) Construction of an OSC with efficient charge injection where the required photon harvest is as high as possible, with transparent electron transport layer (ETL) and hole transport layer (HTL) materials, band arrangement and interface engineering provided. (2) Designing and integrating transparent contact systems that allow efficient charge collection and high AVT instead of opaque contact systems containing thick metals to the OSC. (3) Designing the PC to create the PBG in the structure according to the optical and electrical properties to be improved in the ST-OSC with high AVT and integrating it into the ST-OSC.

In this study, we methodologically based on the three strategies mentioned above and used BHJ in poly (3-hexylthiophene-2, 5-diyl) (P3HT) and poly (6,6-phenyl C61-butyric acid methyl ester) (PCBM)-based inverted structure architecture. We studied the evaluation of optical properties such as AVT, CRI, color, correlated color temperature (CCT), and cell output parameters as a result of 1D-PC integration into ST-OSC. For the first step, we set out from the OSC, examining the cell output parameters and morphological structure in a previous study and determining the optimal structure with high efficiency. In this structure, the highest electrical performance was obtained in the FTO/ZnO/P3HT:PCBM/MoO_3_/Ag opaque-OSC, in which MoO_3_ was chosen as the HTL material^2^. In the second step, with the motivation of determining the optimal structure with high performance, we based our other study on which we obtained ST-OSC with high AVT by modifying the Ag top contact in this OSC with opaque characteristics with MoO_3_/Ag/MoO_3_ (10/d_Ag_/d_MoO3_ nm) DMD transparent top contact. In our related study, we obtained a maximum AVT of 37.42% for the optimal state of d_Ag_ = 6 nm and d_MoO3_ = 30 nm. However, as expected, the PCE decreased to 1.77% with the ST optical characteristic of the structure. In addition, the deficient optical absorption of P3HT:PCBM in the long-wavelength regime of VR allows photons in this region to pass through the structure, and thus the structure is colored red^1^. Therefore, as the third step in the methodology, in this study, the integration of the MgF_2_/MoO_3_ system with the N period to the FTO/ZnO/P3HT:PCBM/MoO_3_/Ag/MoO_3_ ST-OSC was investigated in order to improve the PCE reduced by the ST characteristic and to make the color modification in the structure.

## Results and discussion

In our previous study^1^, we examined the design and optimal values for ST-OSC (without 1D-photonic crystal consists) of P3HT:PCBM active layer (130 nm), ZnO ETL (40 nm), MoO_3_ HTL (40 nm), and MoO_3_/Ag/MoO_3_ (10/d_m_/d_od_ nm) were found to be optimized values. In this study, ST-OSC was examined in order to shift the color coordinates to the Planckian locus and improve the PCE without causing a serious deterioration in the AVTs of the ST-OSC where the PBG was created with 1D-PC. ST-OSC with optimal values were obtained for d_m_ = 6 nm and d_od_ = 30 nm, when based on the highest AVT and photocurrent densities (J_ph_) for the investigated ST-OSC. Although the ST-OSC with a high AVT of 37.42% is experimentally obtained with the integration of DMD into the opaque OSC, the CIE 1931 color coordinates of this structure (CIE x and y are 0.4661 and 0.3587, respectively) are quite far from the Planckian locus. Changing d_m_ slightly shifts the color coordinates to the Planckian locus, but significantly lowers the AVT^1^. Therefore, in this study, we focused on modifying the optical characteristic of the ST-OSC with PBG designed with the MgF_2_/MoO_3_ 1D-PC system, with the aim of shifting the color coordinates to the Planckian locus without causing a serious deterioration in the AVT. The N period MgF_2_/MoO_3_ is denoted by the (MgF_2_/MoO_3_)^N^ label, and the integration of (MgF_2_/MoO_3_)^N^ into the ST-OSC is represented by ST-OSC/(MgF_2_/MoO_3_)^N^. Besides, in terms of examining the electrical performance, in Ag based-DMD transparent contact systems, with an increase of Ag thickness (d_Ag_ = d_m_) from 2 to 12 nm, resistivity and R_sh_ lean from 5.97 × 10^–5^ Ωcm to 0.97 × 10^–5^ Ωcm and from 18.66 Ωsq^−1^ to 2.31 Ωsq^−1^$,$ respectively^34^. Therefore, besides the decrease of AVT with increasing d_Ag_, an improvement in electrical performance may occur.

**Figure 1:**
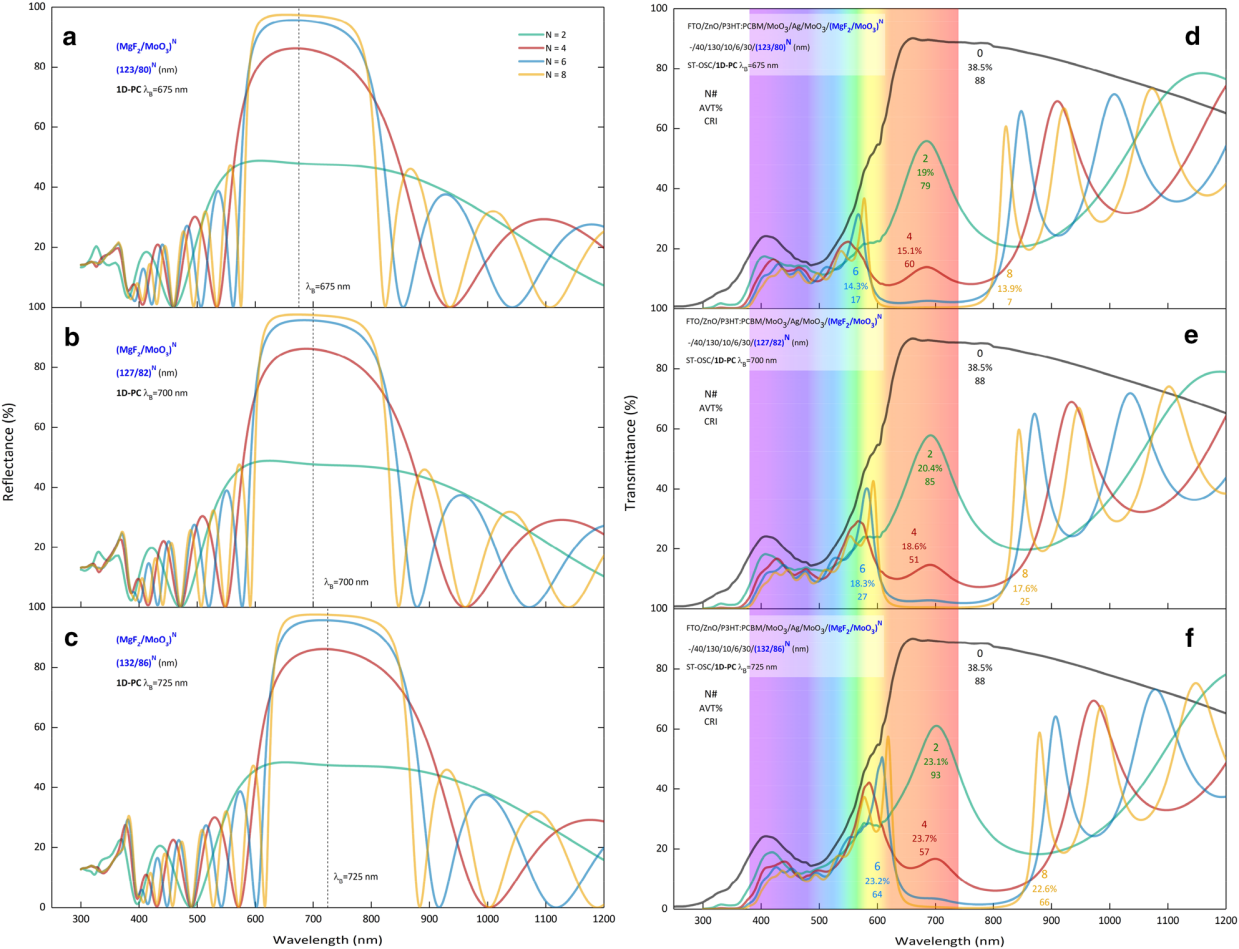
Calculated optical spectra of 1D-Photonic crystal and semi-transparent organic solar cells. Reflection spectra of PBGs designed for N = 2, 4, 6, 8 periods with the MgF_2_/MoO_3_)^N^ 1D-PC system at (**a**) λ_B_ = 675 nm, (**b**) 700 nm, (**c**) 725 nm. Transmission spectra of the ST-OSC/(MgF_2_/MoO_3_)^N^ for N = 2, 4, 6, 8 periods **d** λ_B_ = 675 nm, (**e**) 700 nm, (**f**) 725 nm. The dashed lines in (**a**–**c**) represents the Bragg wavelength set in the calculations. The electromagnetic spectrum is given in the visible region range at d, e, and f. In addition, transmittance spectra of ST-OSC/(MgF_2_/MoO_3_)^N^ for N = 2, 3, 4, 5, 6, 7, and 8 periods are given in Supplementary Fig. S10.

The transmittance of the ST-OSC increases significantly after 600 nm. In this region, the dominance of the $\overline{\mathrm{x} }\left(\uplambda \right)$ color matching function, which is responsible for the red color, makes the ST-OSC color red. Because the optical band gap of P3HT is about 1.9 eV, P3HT:PCBM is intrinsically deep purple-red in color, which makes BHJ almost transparent in the near-infrared (NIR) region^35^. Therefore, the first motivation in theoretical calculations made with TMM is to create a PBG characteristic that will reduce the effectiveness of the color matching function $\overline{\mathrm{x} }\left(\uplambda \right)$ (Supplementary Fig. S3). The center wavelength (λ_B_) corresponding to the resonance wavelength of the PBG to be created with 1D-PC must meet the condition given in Supplementary Eq. (16). PBGs designed using the (MgF_2_/MoO_3_)^N^ 1D-PC system were formed at λ_B_ = 675 nm, 700 nm, 725 nm center wavelengths and N = 2, 4, 6, and 8 periods. Reflection spectra for λ_B_ = 675, 700, 725 nm of the (MgF_2_/MoO_3_)^N^ 1D-PC system with different periods obtained as a result of theoretical calculations are given in Fig. 1a–c, respectively. It is seen that PBG has not formed yet for N = 2 periods, and PBG has formed in the range of 850–554 nm in N = 4 periods, with a width of approximately 300 nm and a reflectance of 49%. With the increase of the period number to N = 8, the PBG characteristic reaches 222 nm width and 97% reflectance in the 806–584 nm range.

Optical spectra of ST-OSC/MgF_2_/MoO_3_)^N^ were calculated by TMM to examine the effect of PBGs of (MgF_2_/MoO_3_)^N^ 1D-PC designed with different parameters on the optical characteristics of ST-OSC. The transmittance spectra of ST-OSC/MgF_2_/MoO_3_)^N^ are presented in Fig. 1d–f for N = 2, 4, 6, 8 periods and λ_B_ = 675, 700, and 725 nm, respectively. In addition, the absorption spectra are given in Supplementary Fig. S4. The addition of (MgF_2_/MoO_3_)^N^ 1D-PC to ST-OSC reduced the transparency of the ST only in the wavelength range where PBG was formed. Since the PBG of the N = 2 (MgF_2_/MoO_3_)^N^ 1D-PC is quite wide, it also reduces the transparency in the VR part. Especially for the N = 4, 6, and 8 periods, there is a significant modification only around 600 nm as desired.

Since the widths of PBGs formed with (MgF_2_/MoO_3_)^N^ 1D-PC are pretty significant, it can lead to a serious deterioration in the transparency of ST-OSCs, if careful λ_B_ is not selected. Even when λ_B_ is selected in the middle of the visible region, the structure becomes completely opaque. Thus, only the low wavelength tail of the PBG can be used when the aim is to modify the transmittance of the ST-OSC while reducing the dominance of the $\overline{\mathrm{x} }\left(\uplambda \right)$ color matching function.

For all ST-OSC/(MgF_2_/MoO_3_)^N^, propagation of the electromagnetic wave in PBG is not allowed. The reflection spectrum formed by PBG corresponds to the wavelength region greater than 600 nm and the IR region. Electromagnetic waves entering the SC from the bottom will be reflected in the active region, with wavelengths that reach the PBG without being absorbed in the active region and are responsible for the PBG. This increases the photocurrent density in ST-OSC and improves cell performance.

**Figure 2:**
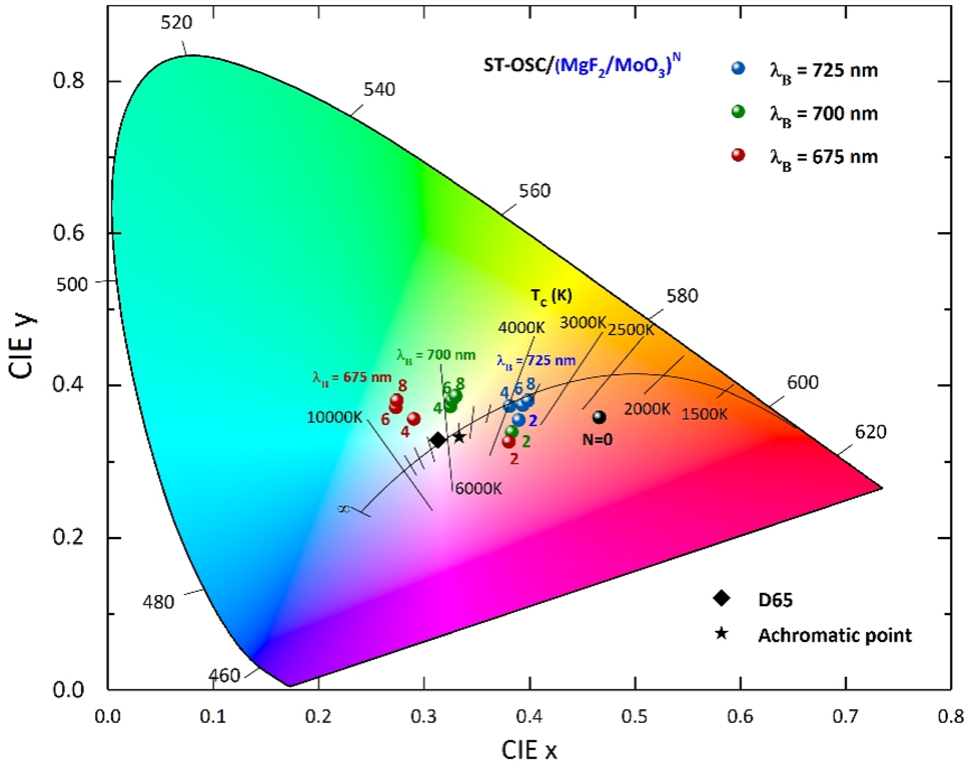
Variation of color coordinates in a semi-transparent organic solar cell. CIE 1931 chromaticity diagram presents the variation of color coordinates for λ_B_ = 675, 700, 725 nm according to the period number of 1D-PC in ST-OSC/(MgF_2_/MoO_3_)^N^. The black line represents the Planckian locus for different color temperatures. The black spherical symbol represents the color coordinate of ST-OSC at N = 0, the black square and star symbolize the D65 and achromatic point locations, respectively.

The variations of the color coordinates of ST-OSC/(MgF_2_/MoO_3_)^N^ with the N for λ_B_ = 675 nm, 700 nm, and 725 nm are given in Fig. 2 (For a more detailed representation, the color coordinates for each λ_B_ relative to N changes are given in Supplementary Fig. S5). When the (MgF_2_/MoO_3_)^N^ 1D-PC 1D-PC system was added to the ST-OSC as intended, a reduction in the CIE 1931 × color coordinate of the structure was achieved with the designed PBG. For λ_B_ = 675 nm, the decrease in CIE x color coordinate with the increase in the number of periods up to 6 in the ST-OSC/(MgF_2_/MoO_3_)^N^ shifts the color of the structure toward the Planckian Locus. The CIE color coordinates for N = 6 and 8 have moved away from the Planckian locus, and their values are almost the same. Because for N = 6 and 8, the decreasing trend in transmittance caused by PBG around 600 nm is almost the same. Further increase in the number of periods does not change the characteristic of the PBG formed for N = 8 and there is no change in the color coordinates. Therefore, agglomeration occurs in a single color coordinate.

The color coordinates for the structure where the PBG is designed as λ_B_ = 675 nm and has N = 4 periods are very close to the Planckian locus, and D65 color coordinates and their values are CIE x = 0.2899 and y = 0.3568. Because the transmittance values of the N = 4 period are almost the same in the 400–700 nm range. This shows that each color matching function affects the structure's color with the same dominance. Therefore, for N = 4, the structure color shifted to the Planckian locus. In addition, PBG designed for N = 4 periods reduces the transparency of the structure much less in VR than in N = 6 and 8 periods in the case of λ_B_ = 675 nm.

For λ_B_ = 700 nm, the decrease in $\mathrm{x}$ color coordinate with the increase in the number of periods up to 4 in ST-OSC/(MgF_2_/MoO_3_)^N^ shifts the color of the structure toward the Planckian Locus. Increasing the period moves the color coordinates slightly away from the Planckian locus. The color coordinates for N = 4, 6 and 8 are almost the same. Because for N = 6 and 8, the decreasing trend in transmittance caused by PBG around 600 nm is almost the same. Further increase in the number of periods does not change the characteristic of the PBG formed for N = 8 and there is no change in the color coordinates. Therefore, agglomeration occurs in single color coordinates. In addition, while the PBG designed for N = 4 reduces the transmittance of the ST-OSC in the wavelength region where the PBG is designed, $\overline{\mathrm{x} }\left(\uplambda \right)$ affects the long-wavelength part of the color matching function. Therefore, PBG is designed for ${\uplambda }_{\mathrm{B}}$=700 nm compared to that designed for λ_B_ = 675 nm makes a more controlled modification of the ST-OSC. Also, PBG is designed for λ_B_ = 700 nm can shift the color of ST-OSC maximally to the Planckian locus.

For the structure in which the PBG is designed as λ_B_ = 700 nm and has N = 4 period, the color coordinates are very close to the Planckian locus and D65 color coordinates, and their values are CIE x = 0.3247 and y = 0.3737. Because the transparency values of the structure with N = 4 periods are almost the same in the 400–700 nm range. This indicates that each color matching function will affect the structure's color with the same dominance. Therefore, the structure color shifted to the Planckian locus for the N = 4 period. In addition, the PBG designed for N = 4 periods has a higher CIE y coordinate if λ_B_ = 700 nm compared to λ_B_ = 675 nm. Because the PBG center is at a high wavelength, it does not affect the wavelengths where $\overline{\mathrm{y} }\left(\uplambda \right)$ is found, which is one of the color matching functions, and the dominance of $\overline{\mathrm{y} }\left(\uplambda \right)$ increases, increasing the value of the y coordinate.

For λ_B_ = 725 nm, the color coordinates are still far from the Planckian Locus, although the CIE x color coordinate decreases with the increase of the period number up to 8 in ST-OSC/(MgF_2_/MoO_3_)^N^. The color coordinates for N = 4, 6 and 8 are almost the same. For N = 4, 6 and 8, the decreasing trend in transmittance caused by PBG around 600 nm is the same and is not in the region where color matching functions are dominant. Therefore, PBG tuned at λ_B_ = 725 nm. It does not shift the color of the ST-OSC/(MgF_2_/MoO_3_)^N^ to the Planckian locus.

**Figure 3:**
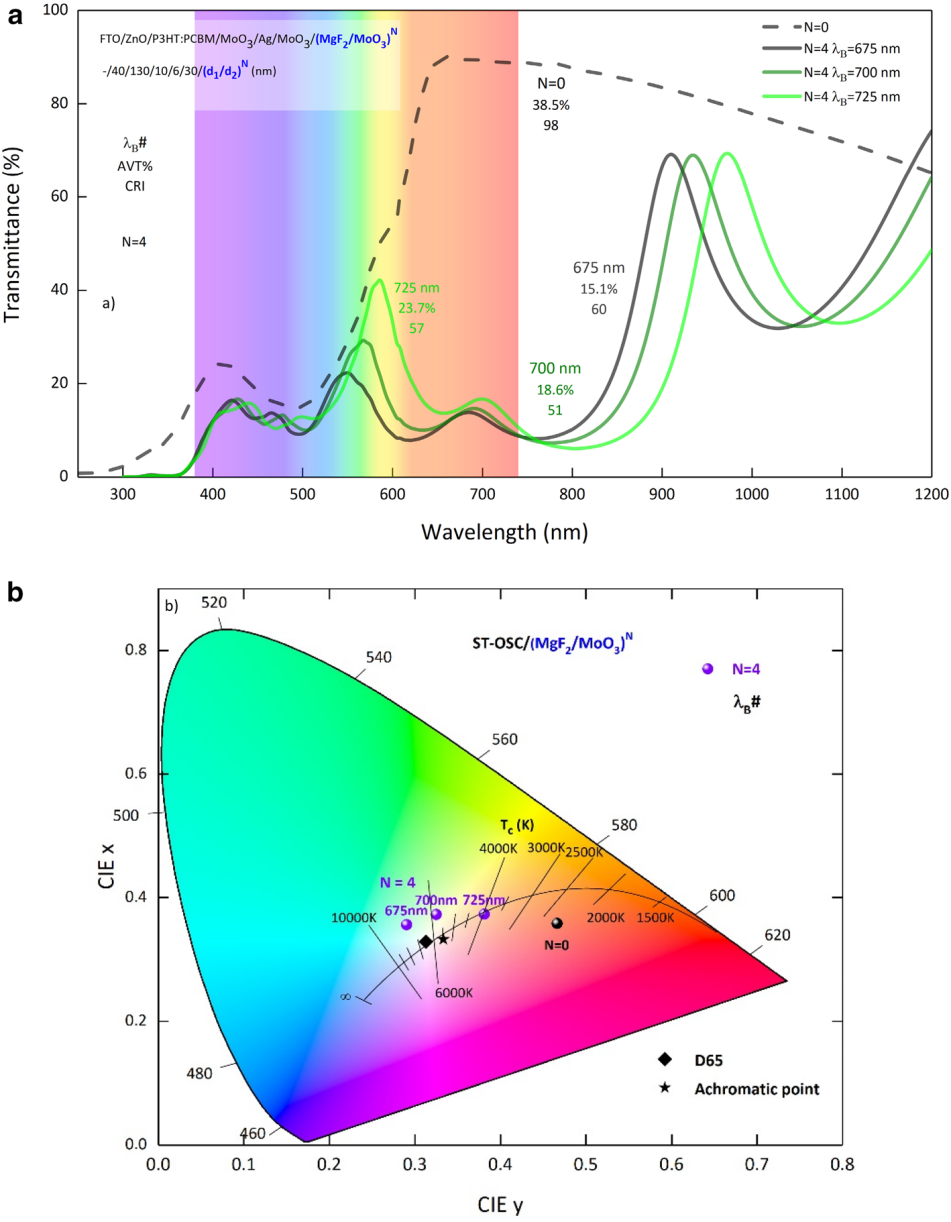
Variation of calculated transmittance spectra and color coordinates of semi-transparent organic solar cell with Bragg wavelength. Representation of (**a**) transmittance spectra and (**b**) color coordinates in CIE 1931 chromaticity diagram for λ_B_ = 725 nm, 700 nm and 675 nm of ST-OSC/(MgF_2_/MoO_3_)^4^ ST-OSC. N = 0 represents the transmittance spectrum and color coordinate of ST-OSC. Symbols representing color coordinates have λ_B_s on them (λ_B_#).

ST-OSC/(MgF_2_/MoO_3_)^N^, as a result of the calculations made by adjusting the period and λ_B_s of PBG, the target of shifting the color coordinates to the Planckian locus has been reached when N = 4 period at λ_B_ = 700 nm and λ_B_ = 625 nm. It is seen that the optimal period for the (MgF_2_/MoO_3_)^N^ 1D-PC system is N = 4. In Fig. 3, the transmittance spectra obtained for different λ_B_s in N = 4 periods are given in Fig. 3a and the variation of color coordinates in Fig. 3b in the CIE 1931 chromaticity diagram. The decrease of λ_B_ from 725 to 675 nm creates a more effective reduction in the wavelength region where the $\overline{\mathrm{x} }\left(\uplambda \right)$ color matching function is dominant in the transmittance spectrum of ST-OSC; thus, the structure color shifts from red to the Planckian locus.

**Figure 4:**
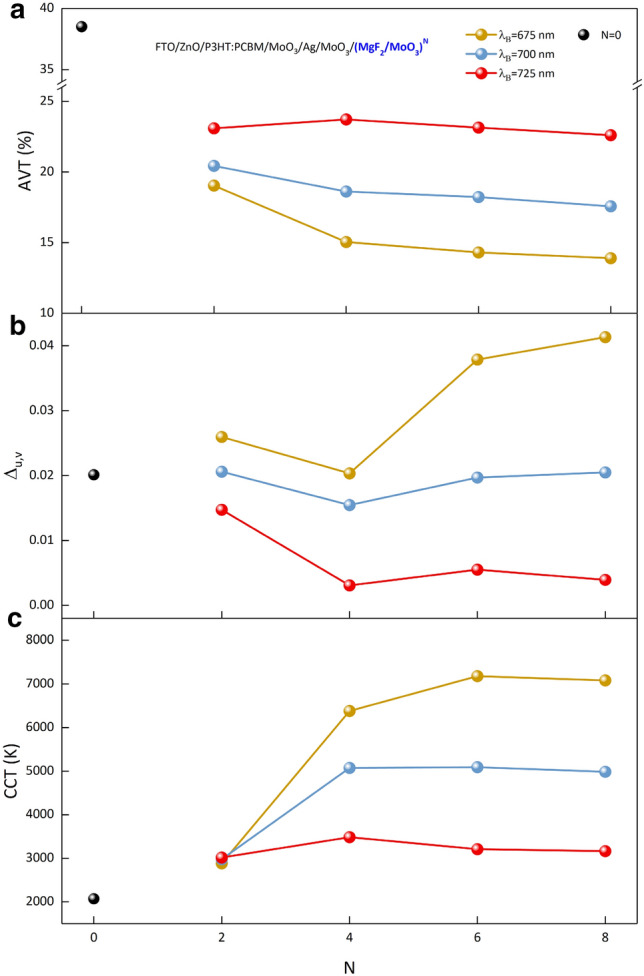
Optical parameters of the semi-transparent organic solar cell. For ST-OSC/(MgF_2_/MoO_3_)^N^, (**a**) AVT, (**b**) Δ_u,v_ and (**c**) CCT changes according to the number of periods at different λ_B_$.$ The black spherical symbol denotes the optical parameters of ST-OSC.

AVTs should be taken as a basis as well as color coordinates for STs. In ST-OSC/(MgF_2_/MoO_3_)^N^, the changes of AVT, Δ_u,v_ and CCT obtained for different λ_B_s according to the period values of PBG of 1D-PC are shown in Fig. 4a–c respectively. The AVT of ST-OSC decreases from 38.52% to 20% as a result of 1D-PC integration. In particular, the highest AVT for each λ_B_ is seen in structures with N = 2 periods, as expected. Because PBGs of structures with N = 2 periods have less reflectivity and reduce the transparency of the structure less than PBGs in other periods.

Considering the effect of the period number on AVT, it is seen that there is no significant change for λ_B_ = 725 nm. However, for λ_B_ = 700 nm and 675 nm, a decrease in AVT occurred in the N = 4 period. Because the low wavelength region of the reflection band of PBG, designed as λ_B_ = 725 nm, did not seriously modify the transmittance spectrum corresponding to the high wavelength region of VR (Supplementary Fig. S6). If the PBG shifts to lower wavelengths, that is, for PBGs designed as λ_B_ = 700 and 675 nm, the optical characteristic of the ST-OSC begins to be affected. This situation causes the AVTs to decrease in case the number of periods increases from N = 2 to 4. In addition, since the shift of λ_B_ to the lower wavelength causes a serious decrease in the transmittance of the ST-OSC, the AVTs are seriously reduced. The AVT for all structures is less than 25%, which is considered the threshold value for window treatments. In addition to this disadvantage, it is a serious design advantage that the structure color shifts to the Planckian locus with a very small number of periods.

Based on the color coordinates that change with the 1D-PC integration of ST-OSCs, besides the optimal period number of the N = 4 period for λ_B_ = 675 nm and 700 nm, it should also be examined which optimal λ_B_ of the N = 4 period in terms of AVTs. For N = 4 periods, the AVT is 15.05% at λ_B_ = 675 nm and 18.63% when λ_B_ = 700 nm. Therefore, when the ST-OSC/MoO_3_/(MgF_2_/MoO_3_)^N^ is designed with PBG with N = 4 period and λ_B_ = 700 nm, it becomes a structure with optimal values based on color coordinates and AVT. Also, the CIE color coordinates x and y for the N = 4 period and λ_B_ = 700 nm are 0.3248 and 0.3733, respectively.

Δ_u,v_ and CCTs, which are essential parameters in the integration of ST-OSCs, especially in equipment that will serve as windows or lighting, should also be examined. The variation of Δ_u,v_ and CCT with N for all designed structures are given in Fig. 4b,c respectively. For the determination of CCT, Δ_u,v_ < 0.0054 is required and this condition is only provided for λ_B_ = 725 nm for N = 4 and 8 periods. At λ_B_ = 725 nm, Δ_u,v_ for N = 4 and 8 periods are 0.0031 and 0.0039 and CCTs are 3483 K and 3161 K, respectively. CCT determination can be made for these structures, but the color coordinates of the structures are quite far from the Planckian locus, as discussed earlier. In cases where the Planckian locus is not taken into account, they can act as light sources with certain CCT and transmitting light on them to structures with relevant parameters.

**Figure 5:**
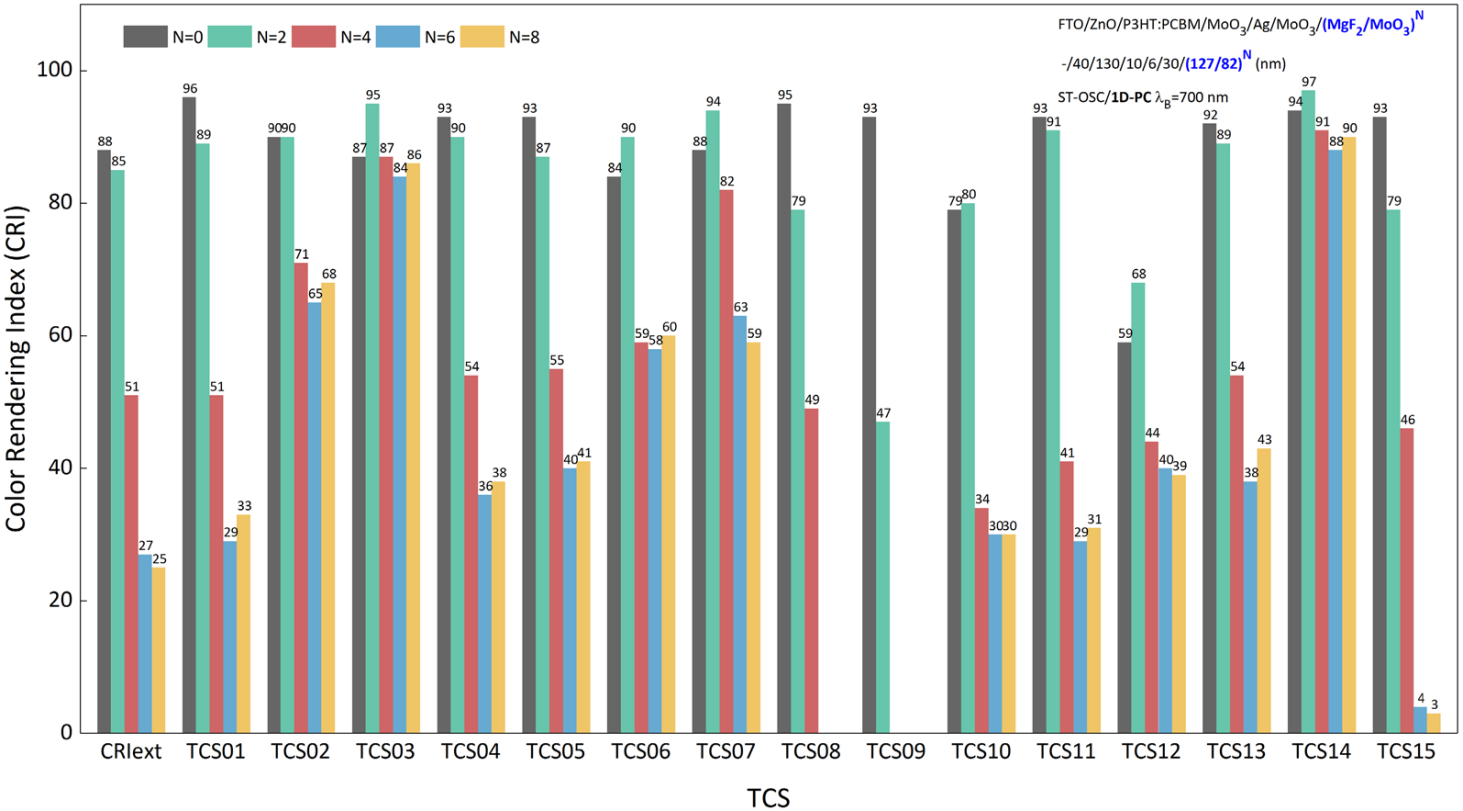
Color rendering characteristic of a semi-transparent organic solar cell. CRI and TCSs for λ_B_ = 700 nm in N = 2, 4, 6, 8 periods in ST-OSC/(MgF_2_/MoO_3_)^N^.

In Fig. 5, CRI is obtained at λ_B_ = 700 nm for different periods (CRI for λ_B_ = 675 nm and 725 nm are presented in Supplementary Fig. S7). The CRI_gen_ and CRI_ext_ for ST-OSC are 91 and 88, respectively. This shows that the color rendering index of ST-OSC, which does not contain 1D-PC and is produced with an AVT of 37.42%, is quite efficient. In addition, the TCS12 coded strong blue color has a CRI of 59. This indicates that the *strong blue* color rendering of the ST-OSC is quite low.

With the addition of 1D-PC to ST-OSC, the CRIs are significantly reduced, as expected. Because the PBG created by 1D-PC reduces the transmittance of ST-OSC at all wavelengths in VR. Therefore, this reduces the CRIs of various colors. In particular, when the PBG reflection intensity of 1D-PC increases with the period, the CRIs of all λ_B_ decrease with the period because the transparency of the ST-OSC decreases.

As a significant decrease, TCS09 coded *strong red* color has a negative CRI at N = 4, 6, and 8 periods. This can be explained by the fact that the small wavelength region of PBG decreases the transmittance up to the region of the red color matching function of ST-OSC. Therefore, due to 1D-PC integration, the red color rendering of the ST-OSC disappears. This shows an inevitable decrease in CRIs when the color of the ST-OSC with 1D-PC shifts to the Planckian locus. Therefore, as optical optimization in ST-OSCs, it is necessary to consider that shifting the color to the desired color coordinates seriously affects the CRI of the structure.

With the aim of shifting the color coordinates of ST-OSCs to the Planckian locus, the optimal values were determined to be λ_B_ = 700 nm and N = 4 periods. In ST-OSC/(MgF_2_/MoO_3_)^N^, the AVT for λ_B_ = 700 nm and N = 4 period is 18.63%, CIE 1931 color coordinates x and y are 0.3248 and 0.3733, respectively. At the same time, we produced ST-OSC and ST-OSC/(MgF_2_/MoO_3_)^N^ under the same growth and deposition conditions for quantitative comparison and conducted an experimental comparative analysis.

**Figure 6:**
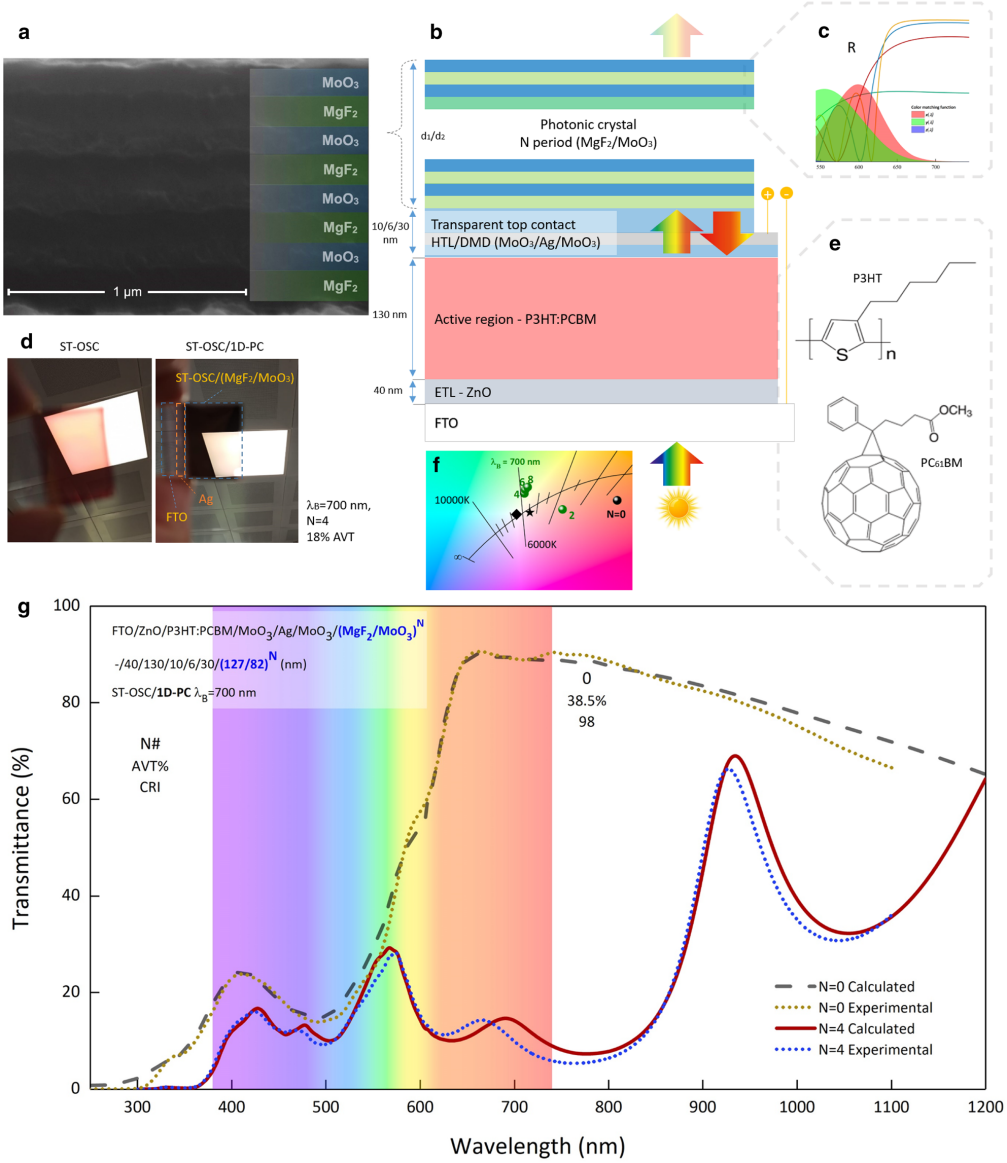
An experimental and contextual demonstration of the produced semi-transparent organic solar cells. (**a**) Cross-section SEM image of (MgF_2_/MoO_3_)^4^ 1D-PC (It is also included in Supplementary Fig. S9). (**b**) The structure of ST-OSC/(MgF_2_/MoO_3_)^4^. (**c**) Transmittance spectra of (MgF_2_/MoO_3_)^N^ and color matching functions. (**d**) Photograph of ST-OSC and ST-OSC/(MgF_2_/MoO_3_)^N^ under lighting. (**e**) Molecular structure of P3HT and PCBM (**f**) color coordinates. (**g**) Calculated and experimental transmittance spectra of ST-OSC(MgF_2_/MoO_3_)^4^ and ST-OSC with λ_B_ = 700 nm PBG.

Figure 6 shows the calculated and experimental transmittance spectra of ST-OSC/MoO_3_/(MgF_2_/MoO_3_)^4^ with λ_B_ = 700 nm PBG and ST-OSC. Calculated and experimental AVTs and CIE 1931 color coordinates are given in Table 1.

**Table 1:** Comparison of optical characteristics obtained by experiment and calculation.

	AVT (%)	CIE $x$	CIE $y$
Calculated	18.63	0.3248	0.3733
Experimental	17.98	0.3328	0.3672

Calculated and experimental AVT and CIE 1931 color coordinates of ST-OSC/(MgF_2_/MoO_3_)^4^ ST-OSC with λ_B_ = 700 nm PBG.

There are slight differences in some regions in the calculated and experimental transmittance spectra of ST-OSC containing 1D-PC given in Fig. 6g. This may have resulted from the thickness calibration of the layers that make up the 1D-PC in the sputter system and the deviation in the targeted thicknesses of the layers that make up the ST-OSC. Especially since the PBG characteristic of (MgF_2_/MoO_3_)^4^ is highly dependent on d_1_ and d_2_, a slight deviation in the thickness of these layers from the targeted values changes the optical characteristic considerably. In addition, the chemical structure of the P3HT:PCBM active region is susceptible to ambient conditions. This may cause the active area not to be covered in the desired thickness and homogeneity. Therefore, there is a difference in the experimental and calculated transmittance spectra. However, this did not cause a difference in the AVT and CIE 1931 color coordinates values given in Table 1. The compatibility between the calculation and the experiment shows that the TMM calculations made within the scope of the study give excellent results.

**Figure 7:**
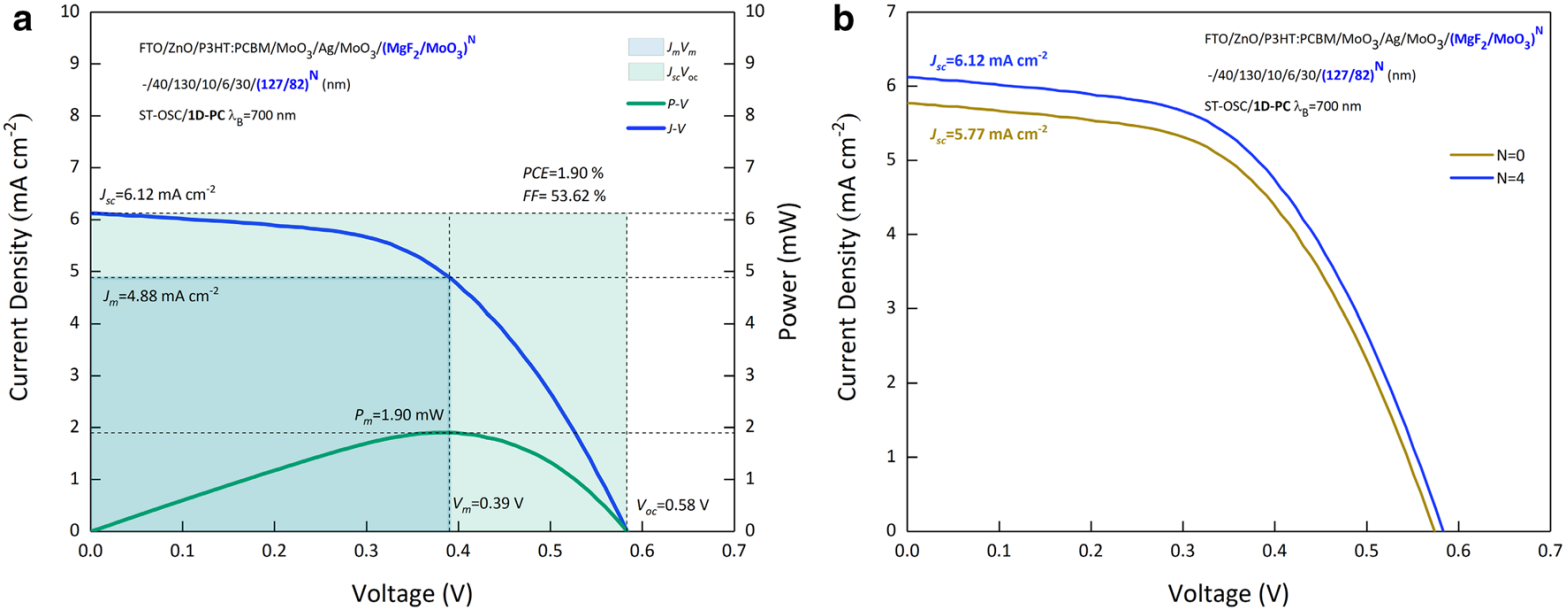
Current Density–Voltage characteristics. (**a**) J–V and P–V characteristics of ST-OSC/(MgF_2_/MoO_3_)^4^ with λ_B_ = 700 nm PBG. (**b**) Comparative J–V characteristics of ST-OSC and ST-OSC/(MgF_2_/MoO_3_)^4^.

The current density–voltage (J-V) and Power-Voltage (P–V) characteristics of ST-OSC/(MgF_2_/MoO_3_)^4^ with λ_B_ = 700 nm PBG, whose optimal values were determined and produced are given in Fig. 7a. In Fig. 7b, the J-V characteristics of ST-OSC and ST-OSC/(MgF_2_/MoO_3_)^4^ are presented comparatively. In addition, the photovoltaic performance of ST-OSC/(MgF_2_/MoO_3_)^4^, whose optical parameters were determined by TMM calculations and produced at optimal values, and opaque-OSC and ST-OSC, which we examined in our previous study^1^, are given in Table 2.

**Table 2:** Photovoltaic performance of solar cells.

	J_sc_ (mA cm^−2^)	J_m_ (mA cm^−2^)	V_oc_ (V)	V_m_ (V)	P_m_ (mW)	FF (%)	PCE (%)
ST-OSC ^1^	5.77	4.73	0.57	0.37	1.77	53.21	1.77
ST-OSC/1D-PC	6.12	4.88	0.58	0.39	1.90	53.61	1.90
Opaque-OSC ^1^	6.72	4.81	0.59	0.42	2.02	51.00	2.02

An electrical evaluation of MoO_3_/Ag/MO_3_ (10/6/30 nm), used as a transparent contact system in ST-OSC structures presented in Table 2, can be done based on the literature. The roughness and thickness of ultra-thin Ag films can severely affect the resistivity and layer strength. This effect is due to the deposition nature of Ag, especially in physical vapor deposition techniques. It is usual for Ag particles to accumulate on islands during the deposition process. This creates a negative effect on the movement of electrons in the metal layer and requires investigation of different transport mechanisms. For this reason, ultra-thin, ultra-smooth, continuous, low-loss and low-R_sh_ Ag thin films can be produced by improving sputtering parameters in the literature^34,36^. Especially in the sputtering system, by increasing the sputtering time for Ag and reducing the gaps between Ag islands, R_sh_ for Ag thin film up to 4 nm can be significantly reduced^34^. For d_Ag_ > 4 nm, the R_sh_ decreases and becomes around 4 Ωsq^−1^ in d_Ag_ = 6 nm^34^. Therefore, the MoO_3_/Ag/MO_3_ (10/6/30 nm) also has very convenient contact properties for electrical performance^15^.

The FFs of ST-OSC/(MgF_2_/MoO_3_)^4^ with λ_B_ = 700 nm PBG are 53.61%. For opaque OSC, ST-OSC and ST-OSC/(MgF_2_/MoO_3_)^4^, HTL is MoO_3_ and d_id_ is 10 nm as well. The band arrangement and contact structures of the structures are the same. This does not cause a severe change in V_oc_, J_m_ and V_m_. However, J_sc_ for ST-OSC/(MgF_2_/MoO_3_)^4^ is higher than for ST-OSC and less than for the opaque one. Re-absorption process occurs as a result of the reflection of photons in the energy remaining in the PBG of the (MgF_2_/MoO_3_)^4^ 1D-PC, which enters by the FTO in the OSC and is not absorbed in the P3HT:PCBM active layer, back into the structure. The increase in absorption produces an increase in J_sc_. Thanks to the DMD transparent top contact in the ST-OSC, low reflection and high transmittance do not support the re-absorption process. Therefore, the lowest J_sc_ and PCE are observed in ST-OSC. In opaque OSC, thick Ag contact reflects all photons in the energy responsible for AM1.5G spectral distribution and (MgF_2_/MoO_3_)^4^ 1D-PC photons in the PBG range back into the structure. Therefore, the ST-OSC containing 1D-PC only reabsorbs photons in the PBG region. Therefore, the J_sc_ and PCEs obtained for ST-OSC/(MgF_2_/MoO_3_)^4^ are lower than the values observed in the opaque-OSC. In light of all these inferences, when the (MgF_2_/MoO_3_)^4^ 1D-PC system is designed to have λ_B_ = 700 nm PBG and N = 4 period, the integration into ST-OSC enables the modification of optical properties and shifting of the color coordinates to the Planckian locus. In addition, it is seen that it improves cell output parameters.

## Material and methods

### Calculation of optical spectra

We performed the calculation of optical spectra (MgF_2_/MoO_3_)^N^ and ST-OSC/(MgF_2_/MoO_3_)^N^ by adapting the such as absorption, transmittance and reflection spectral data in the Transfer matrix method (TMM), which is a very effective method used in the simulations of optoelectronic devices. TMM is a powerful method that analyzes how the electromagnetic wave propagates in the structure and theoretically determines the optical characteristics of the structure, especially in structures such as DMD and PC, where different dielectric media are grown on top of each other^1,15^. The method followed and the equations used in the calculations made with TMM are given in detail in our previous study^1^ and Supplementary Information.

### Average visible transmittance

The transparency properties of optoelectronic systems are evaluated by AVTs and determined by the transmittance characteristics in the visible light wavelength range (370 nm–740 nm), taking into account the photonic response of the human eye. For AVT calculations, transmittance spectra of ST-OSC/(MgF_2_/MoO_3_)^N^ calculated by TMM were used. The method followed in the calculations and the equations used are given in detail in our previous study^1^ and Supplementary Information.

### Calculation of CIE 1931 color coordinates

Another characteristic of ST-OSCs as important as AVT is the color coordinates (x, y) in the CIE 1931 chromaticity diagram. The CIE 1931 chromaticity diagram is designed based on the photonic response of the human eye and is often used to determine the color properties of illuminators. The method followed and the equations used to calculate the CIE 1931 color coordinates of ST-OSC/(MgF_2_/MoO_3_)^N^ are in detail in our previous study^1^ and Supplementary Information.

### Color rendering index

Color rendering index (CRI) is a measure of how faithful the color of an object is when it is illuminated with an ideal or natural light source^37^. Therefore, CRI creates a measurement system that determines whether the colors of illuminated objects are true to their originality and takes values in the 0–100. A CRI of 100 can be seen in standardized daylight sources or incandescent lamp bodies. CRIs of 90 and above are considered excellent, while CRIs below 80 are generally considered poor^37,38^. The method followed and the equations used to calculate the CIEs of ST-OSC/(MgF_2_/MoO_3_)^N^ are in detail in Supplementary Information.

### Correlated color temperature

Correlated color temperature (CCT) is an absolute and one-dimensional measurement system that describes a specific point along the blackbody curve in the CIE 1931 chromaticity diagram. It is an important criterion used to define mainly white light sources. CCT also emerges as a system that shows the similarity of optoelectronic devices with transparent or semi-transparent characteristics to light emitters in various technological applications. The method followed and the equations used to calculate the CCTs of ST-OSC/(MgF_2_/MoO_3_)^N^ are in detail in the Supplementary Information.

### Experimental details

The active region, HTL and ETL of the ST-OSC/1D-PC designed and produced within the scope of this study and the opaque-OSC and ST-OSCs that we examined in our previous study are the same. The active region consists of a mixture of Poly (3-hexylthiophene-2, 5-diyl) (P3HT) and poly (6, 6-phenyl C61-butyric acid methyl ester) (PCBM). OSCs are in the inverted device architecture and bulk-heterojunction (BHJ) structure. All layers except the active region in the OSCs were coated using the Nanovak NVTS500 Sputtering system. The fabrication steps and structure parameters for OSCs are detailed in our previous study^1,2^.

In this study, the optical characteristic of FTO/ZnO/P3HT:PCBM/MoO_3_/Ag/MoO_3_ ST-OSC was modified with PBG formed with (MgF_2_/MoO_3_)^N^ 1D-PC system. (MgF_2_/MoO_3_)^N^ 1D-PCs PBG is determined according to the Bragg wavelength, which corresponds to the resonance wavelength and is the center wavelength, and is adjusted according to the optical property desired to be changed in ST-OSC. Cross-section SEM image of (MgF_2_/MoO_3_)^N^ 1D-PC system and schematic representation of ST-OSC/(MgF_2_/MoO_3_)^N^ are given in Fig. 6a,b respectively.

The MgF_2_ and MoO_3_ layers constituting the 1D-PC were deposited using the sputtering technique. As mentioned in the Result and Discussion section, the design parameters were calculated theoretically according to the determined period and λ_B_ based on the optical properties to be changed, and the structures were produced. In the (MgF_2_/MoO_3_)^N^ 1D-PC system, the thickness of the first layer, MgF_2_, is d_1_ and the thickness of the second layer, MoO_3_, is d_2_. By using Supplementary Eq. (16), the d_1_ and d_2_ are adjusted according to the characteristic of the PBG to be designed. The d_1_ and d_2_ thicknesses calculated for each λ_B_ are given in Table3.

**Table 3:** The 1D-PC structural parameters.

λ_B_ (nm)	675	700	725
d_1_(nm)	123	127	132
d_2_ (nm)	80	82	86

(MgF_2_/MoO_3_)^N^ 1D-PC system thicknesses d_1_ and d_2_ for λ_B_ = 675, 700, 725 nm.

The dielectric constant of (MgF_2_/MoO_3_)^N^ 1D-PC—hence the refractive index—changes periodically in only one dimension. In this respect, the structure is actually a dielectric mirror structure and the width and reflection intensity of the PBG formed by (MgF_2_/MoO_3_)^N^ 1D-PC are related to the refractive index of each layer. In addition, it is desired that the absorption of MgF_2_ and MoO_3_ is very low in the wavelength range where PBG is desired to be formed, and if possible, it should not be. The changes of refractive indices (n) and extinction coefficients (k_ex_) of MgF_2_ and MoO_3,_ depending on wavelength, are given in Supplementary Fig. S8.

The difference between the n of MgF_2_ and MoO_3_ provides the periodic refractive index distribution in the (MgF_2_/MoO_3_)^N^ 1D-PC. This high n difference allows higher reflectance to be obtained in PBG with less period numbers. Therefore, with (MgF_2_/MoO_3_)^N^ 1D-PC, it is possible to design lighter and more flexible structures with desired optical properties by using less material. Especially at wavelengths higher than 600 nm, the dependence of the n of both MgF_2_ and MoO_3_ on wavelength disappears. This situation causes the PBG to be formed at wavelengths larger than 600 nm to be sharper and the PBG characteristic to be less dependent on the wavelength. Especially since the optical design is made through transmittance and reflection in STs, it is aimed that the absorption of the materials to be used in 1D-PCs to be integrated into STs be as low as possible. The k_ex_ of MgF_2_ is very close to zero for all wavelengths, indicating the suitability of MgF_2_ for use in 1D-PCs. The k_ex_ of MoO_3_ approaches zero after 500 nm and is suitable for PBG designs with wavelengths greater than 500 nm.

Optical characteristics of the fabricated structures were determined with Perkin Elmer Lambda 2S UV–Vis–NIR spectrometer in the wavelength range of 300–1100 nm. In addition, Keithley 4200 source meter was used for J-V measurements and Newport Oriel-Sol1A solar simulator was used for AM 1.5G illumination. Cross-section SEM image of 1D-PC in ST/(MgF_2_/MoO_3_)^N^ was taken with FIE Quanta FEG 450.

## Conclusion

In summary, with the fine-tuned 1D-PC integration into ST-OSC, we were able to shift the ST-OSC color, which is dark purple-red by nature of the organic semiconductor that forms the active region, to the Planckian locus, as well as improve PCE. Methodologically, we investigated the integration of the (MgF_2_/MoO_3_)^N^ 1D-PC system into the P3HT:PCBM-based ST-OSC to improve the ST-reduced absorption characteristic and thus the PCE and for color modification in ST-OSC. Optical characteristics and AVT, CRI, color, and CCT ​​of ST-OSCs designed with the integration of fine-tuned (MgF_2_/MoO_3_)^N^ 1D-PC system at different periods and λ_B_ were calculated with light management engineering-based approaches. Each optical characteristic is examined in detail and the potential of using the structures formed as a result of the 1D-PC integration of ST-OSC in architectural and industrial designs that require high optical performance is discussed. In the work output, the color coordinates of the ST-OSC could be shifted to the Planckian locus and D65 color coordinates with the 1D-PC designed for the N = 4 period and λ_B_ = 700 nm without causing a serious deterioration in the AVTs. CIE x and y are 0.3248 and 0.3733, respectively, and the AVT for this structure was obtained as 18.63%. In addition, an improvement in the photovoltaic performance of ST-OSC was observed with the (MgF_2_/MoO_3_)^N^ 1D-PC system. Finally, in the study, it was determined that the integration of 1D-PC into ST-OSC improves cell output parameters as well as modifying optical properties and shifting color coordinates to the Planckian locus.

## Supplementary Information

Supplementary Information.

## Data Availability

The materials and data that support the findings of this study are available from the corresponding authors on request.

## References

[1] Çetinkaya Ç (2021). Design and fabrication of a semi-transparent solar cell considering the effect of the layer thickness of MoO_3_/Ag/MoO_3_ transparent top contact on optical and electrical properties. Sci. Rep..

[2] Çetinkaya Ç (2021). Evaluation on output parameters of the inverted organic solar cells depending on transition-metal-oxide based hole-transporting materials. Opt. Mater..

[3] Brus VV (2019). Solution-processed semitransparent organic photovoltaics: From molecular design to device performance. Adv. Mater..

[4] Kaltenbrunner M (2012). Ultrathin and lightweight organic solar cells with high flexibility. Nat. Commun..

[5] Zhang Y-X (2019). Synergetic transparent electrode architecture for efficient non-fullerene flexible organic solar cells with> 12% efficiency. ACS Nano.

[6] Schopp N, Brus VV, Nguyen TQ (2021). On optoelectronic processes in organic solar cells: From opaque to transparent. Adv. Opt. Mater..

[7] Liu Q (2020). 18% Efficiency organic solar cells. Sci. Bull..

[8] Li S, Li C-Z, Shi M, Chen H (2020). New phase for organic solar cell research: Emergence of Y-series electron acceptors and their perspectives. ACS Energy Lett..

[9] Hu Y (2018). Effect of alkyl-chain length on charge transport properties of organic semiconductors and organic field-effect transistors. Adv. Electron. Mater..

[10] Cheng P, Li G, Zhan X, Yang Y (2018). Next-generation organic photovoltaics based on non-fullerene acceptors. Nat. Photonics.

[11] Seo K, Lee J, Jo J, Cho C, Lee J (2019). Highly efficient (> 10%) flexible organic solar cells on PEDOT-free and ITO-free transparent electrodes. Adv. Mater..

[12] Xu G (2017). High-performance colorful semitransparent polymer solar cells with ultrathin hybrid-metal electrodes and fine-tuned dielectric mirrors. Adv. Funct. Mater..

[13] Zhang Y (2021). Efficient semi-transparent organic solar cells with high color rendering index enabled by self-assembled and knitted AgNPs/MWCNTs transparent top electrode via solution process. Adv. Opt. Mater..

[14] Lunt RR (2012). Theoretical limits for visibly transparent photovoltaics. Appl. Phys. Lett..

[15] Çetinkaya Ç, Çokduygulular E, Güzelçimen F, Kınacı B (2022). Functional optical design of thickness-optimized transparent conductive dielectric-metal-dielectric plasmonic structure. Sci. Rep..

[16] Çetinkaya Ç (2022). Highly improved light harvesting and photovoltaic performance in CdTe solar cell with functional designed 1D-photonic crystal via light management engineering. Sci. Rep..

[17] Çetinkaya Ç (2022). Efficient and high-bifacial CdTe-based solar cell enabled by functional designed dielectric/metal/dielectric transparent top contact via light management engineering. Opt. Mater..

[18] Wu J-S, Cheng S-W, Cheng Y-J, Hsu C-S (2015). Donor–acceptor conjugated polymers based on multifused ladder-type arenes for organic solar cells. Chem. Soc. Rev..

[19] Maniyara RA, Mkhitaryan VK, Chen TL, Ghosh DS, Pruneri V (2016). An antireflection transparent conductor with ultralow optical loss (<2 %) and electrical resistance (<6 Ω sq−1). Nat. Commun..

[20] Yeh T-H (2018). Vacuum-deposited MoO_3_/Ag/WO_3_ multilayered electrode for highly efficient transparent and inverted organic light-emitting diodes. Org. Electron..

[21] Drolet, N. Organic photovoltaic: Efficiency and lifetime challenges for commercial viability. In *2012 MRS Spring Meeting & Exhibit, San Francisco, CA, Moscone West Convention Center, Marriott Marquis* (2012).

[22] Betancur R (2013). Transparent polymer solar cells employing a layered light-trapping architecture. Nat. Photonics.

[23] Ramírez Quiroz CO (2016). Coloring semitransparent perovskite solar cells via dielectric mirrors. ACS Nano.

[24] Li Y (2021). High-performance semi-transparent organic photovoltaic devices via improving absorbing selectivity. Adv. Energy Mater..

[25] Zhang D-D (2013). Enhanced performance of semitransparent inverted organic photovoltaic devices via a high reflector structure. ACS Appl. Mater. Interfaces.

[26] Zhang Y (2016). Colorful semitransparent polymer solar cells employing a bottom periodic one-dimensional photonic crystal and a top conductive PEDOT:PSS layer. J. Mater. Chem. A.

[27] Liu W, Ma H, Walsh A (2019). Advance in photonic crystal solar cells. Renew. Sustain. Energy Rev..

[28] Lova P, Manfredi G, Comoretto D (2018). Advances in functional solution processed planar 1D photonic crystals. Adv. Opt. Mater..

[29] Zheng W (2020). Efficient low-cost all-flexible microcavity semitransparent polymer solar cells enabled by polymer flexible one-dimensional photonic crystals. ACS Appl. Mater. Interfaces.

[30] Zhenhua W, Simin L, Wentao Z (2016). Back reflector of solar cells consisting of one-dimensional photonic crystal and double-layered two-dimensional photonic crystal. Acta Photonica Sin..

[31] Chang SY, Cheng P, Li G, Yang Y (2018). Transparent polymer photovoltaics for solar energy harvesting and beyond. Joule.

[32] Xia R, Brabec CJ, Yip H-L, Cao Y (2019). High-throughput optical screening for efficient semitransparent organic solar cells. Joule.

[33] Colodrero S (2009). Porous one-dimensional photonic crystals improve the power-conversion efficiency of dye-sensitized solar cells. Adv. Mater..

[34] Zhang D (2011). Effect of silver evolution on conductivity and transmittance of ZnO/Ag thin films. J. Appl. Phys..

[35] Chang J-K (2020). Solution-processed, semitransparent organic photovoltaics integrated with solution-doped graphene electrodes. Sci. Rep..

[36] Chen W, Thoreson MD, Ishii S, Kildishev AV, Shalaev VM (2010). Ultra-thin ultra-smooth and low-loss silver films on a germanium wetting layer. Opt. Express.

[37] Ohta N, Robertson AR (2005). Colorimetry: Fundamentals and Applications.

[38] Li Z, Ma T, Yang H, Lu L, Wang R (2021). Transparent and colored solar photovoltaics for building integration. Solar RRL.

