# Supplementary material for: Enhancement of color and photovoltaic performance of semi-transparent organic solar cell via fine-tuned 1D photonic crystal
Source: Sci Rep. 2022 Nov 12;12:19400. doi: 10.1038/s41598-022-24113-9 (PMC9653454; doi:10.1038/s41598-022-24113-9)
Supplement: Supplementary file 1 — Supplementary Information. [file 41598_2022_24113_MOESM1_ESM.docx]

**Supplementary Information**

**Enhancement of Color and Photovoltaic Performance of
Semi-Transparent Organic Solar Cell via Fine-Tuned 1D Photonic Crystal**

Çağlar Çetinkaya^1*^, Erman Çokduygulular^2^, Barış Kınacı^3,4^, Serkan Emik^5^, Nihan Akın Sönmez^3,4^, Süleyman Özçelik^3,4^

### ^1^Physics Department, Faculty of Science, Istanbul University, TR-34134, Istanbul, Turkey

### ^2^Department of Engineering Sciences, Faculty of Engineering, Istanbul University-Cerrahpaşa, TR-34320, Istanbul, Turkey

### ^3^Department of Photonics, Faculty of Applied Sciences, Gazi University, TR-06500, Ankara, Turkey

### ^4^Photonics Application and Research Center, Gazi University, TR-06500, Ankara, Turkey

### ^5^Department of Chemical Engineering, Faculty of Engineering, Istanbul University-Cerrahpaşa, TR-34320, Istanbul, Turkey

*^*^Correspondence should be addressed to Çağlar Çetinkaya. (email:* [*caglarcetinkaya@istanbul.edu.tr*](mailto:baris.kinaci@istanbul.edu.tr)*)*

**Calculation of Optical Spectra**

We performed the calculations using the TMM, a highly effective method used in the simulations of optoelectronic devices. TMM is one of the important methods that analyze how the electromagnetic wave propagates within the structure and theoretically determine the optical characteristics of the structure^1–3^. The position of the electric and magnetic field components within the DMD or ST-OSC can be determined by a transfer matrix and propagation matrix^1^. While the electric and magnetic field components of the electromagnetic wave are connected with a transfer matrix at each interface of each layers, the spreading field components in the layers are connected to each other by the propagation matrix.

| 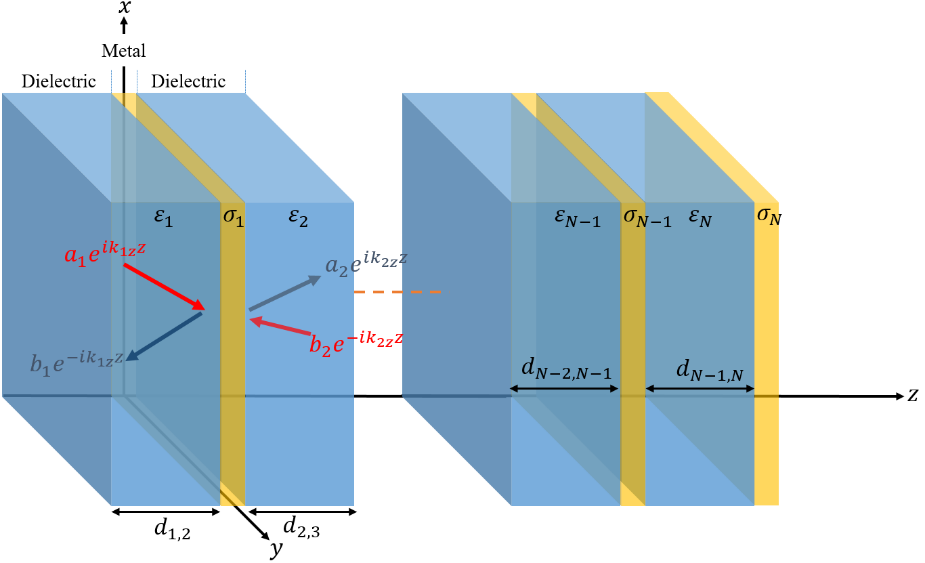 |
| --- |
| **Supplementary Figure 1.** A thin metal layer with σ conductivity is surrounded by two dielectric layers with values $\varepsilon_{1}$ and $\varepsilon_{2}$. The structure is self-repeatable and can be reduced to a DMD structure for a single metal layer. Red and blue arrows represent the electromagnetic wave at the interface and the reflected, respectively. |

As seen in Supplementary Figure 1, the metal layer surrounded by two dielectrics for the DMD structure has a conductivity ($\sigma$) distributed parallel to the $z=0$planes. It is necessary to examine the propagation of the electromagnetic wave on the interfaces of the conductor with the dielectrics. Assuming that the electromagnetic wave is polarized in the $y$ direction and propagates in the $z$ direction, the $s$ and $p$ polarizations can be examined. The magnetic field for polarization *p* can be written in the following form:

| $H_{1y}=\alpha_{1}e^{i\vec{k}_{1}.\vec{r}}+\beta_{1}e^{i\vec{k}_{1}.\vec{r}}=\left( \alpha_{1}e^{ik_{1z}z}+\beta_{1}e^{-ik_{1z}z} \right)e^{-ik_{1x}x}, z<0$ | (1) |
| --- | --- |
| $H_{2y}=\alpha_{2}e^{i\vec{k}_{2}.\vec{r}}+\beta_{2}e^{i\vec{k}_{2}.\vec{r}}=\left( \alpha_{2}e^{ik_{2z}z}+\beta_{2}e^{-ik_{2z}z} \right)e^{-ik_{2x}x}, z>0$ | (2) |

where $\vec{k}_{i}=\sqrt{\varepsilon_{i}}\frac{\omega}{c} (i=1, 2)$ is the wave vector of the electromagnetic wave, $\varepsilon_{i} (i=1, 2)$ is the dielectric constant of the medium, $\omega$ is the angular frequency, $c$ is the propagation velocity of the electromagnetic wave in space and $\alpha_{i}$ and $\beta_{i} (i=1,2)$ are the coefficients. According to Snell’s law, the $x$ components of the wave vector in both media at the interface will be equal to each other: $k_{1x}=k_{2x}$. If we also apply the boundary conditions of the electric field and the magnetic field for the interface^4^, we obtain the equations found below:

| $\left. \hat{n}_{s}\times\left( \vec{E}_{2}-\vec{E}_{1} \right) \right\vert_{z=0}=0$ | (3) |
| --- | --- |
| $\left. \hat{n}_{s}\times\left( \vec{H}_{2}-\vec{H}_{1} \right) \right\vert_{z=0}=\vec{J}$ | (4) |

Here, $\hat{n}_{s}$ is the normal unit vector of the surface, and $\vec{J}$ is the surface current density of the metallic layer. In addition, by obtaining $\vec{J}$ by Ohm’s law and applying the $z=0$ condition, the following equations are obtained:

| $\frac{k_{1z}}{\varepsilon_{1}}\left( a_{1}-b_{1} \right)-\frac{k_{2z}}{\varepsilon_{2}}\left( a_{2}-b_{2} \right)=0$ | (5) |
| --- | --- |
| $\left( a_{1}+b_{1} \right)-\left( a_{2}+b_{2} \right)=J_{x}$ | (6) |
| $J_{x}=\left. \sigma E_{x} \right\vert_{z=0}=\frac{\sigma k_{2z}}{\varepsilon_{0}\varepsilon_{2}\omega}\left( a_{2}-b_{2} \right)$ | (7) |

where $\varepsilon_{0}$ is the permittivity of the space. By combination of equations (5), (6) and (7), $a_{i}$ and $b_{i}$ ($i=1$) can be associated with $a_{i+1}$, $b_{i+1}$ and the transition matrix $M_{i\to i+1}$.

| $\binom{a_{i}}{b_{i}}=M_{i\to i+1}\binom{a_{i+1}}{b_{i+1}}$ | (8) |
| --- | --- |

The $M_{i\to i+1}$ transition matrix is ​​$M_{1\to2}$ as follows when$i=1$ is selected for DMD design with a single metallic layer:

| $M_{1\to2}=\frac{1}{2}\left( \begin{matrix} 1+n_{p}+\xi_{p} & 1-n_{p}-\xi_{p} \\ 1-n_{p}+\xi_{p} & 1+n_{p}-\xi_{p} \end{matrix} \right)$ | (9) |
| --- | --- |

where $n_{p}=\frac{\varepsilon_{1}k_{2z}}{\varepsilon_{2}k_{1z}}$, and $\xi_{p}=\frac{\sigma k_{2z}}{\varepsilon_{0}\varepsilon_{2}\omega}$. The above magnetic field and related boundary conditions for polarization $p$ and all processes performed by the application of Ohm’s law are also applicable to the electric field component for *s* polarization. In this case, again by using Ohm’s law and boundary conditions, the transition matrix of *s* polarization is obtained as follows:

| $M_{1\to2}=\frac{1}{2}\left( \begin{matrix} 1+n_{s}+\xi_{s} & 1-n_{s}+\xi_{s} \\ 1-n_{s}-\xi_{s} & 1+n_{s}-\xi_{s} \end{matrix} \right)$ | (10) |
| --- | --- |

where $\mu_{0}$ is the transmittance of the space, the parameters $n_{s}$ and $\xi_{s}$ are equal to the terms $\frac{k_{2z}}{k_{1z}}$ and $\frac{\sigma\mu_{0}\omega}{k_{1z}}$, respectively. The $n_{p}$ and $n_{s}$ terms used in the derivation of the equations are directly related to the refractive index of the layers and include the absorption coefficient within the complex term. These values ​​depend on the wavelength and the angle of incidence of the electromagnetic wave.

Considering the equations, the transition matrices obtained for both $s$ and $p$ polarizations are the same, except for the sign difference in non-diagonal components. By making the necessary arrangements for $j=(s,p)$ and $\eta_{p}=1$,$\eta_{s}=-1$, a common transition matrix can be parameterized as follows:

| $M_{1\to2}=\frac{1}{2}\left( \begin{matrix} 1+n_{j}+\xi_{j} & 1-n_{j}-{\eta_{j}\xi}_{j} \\ 1-n_{j}+{\eta_{j}\xi}_{j} & 1+n_{j}-\xi_{j} \end{matrix} \right)$ | (11) |
| --- | --- |

The relationship between the transition ($t$) and reflection ($r$) coefficients of the electromagnetic wave at the interfaces with the transition matrix as well as the transmittance ($T$) and reflection ($R$) spectrum of the DMD and can be calculated as follows:

| ${R= \left\vert r \right\vert}^{2}=\left\vert\frac{M_{2\to1}}{M_{1\to1}} \right\vert^{2}$ | (12) |
| --- | --- |
| ${T= \left\vert t \right\vert}^{2}=\left\vert\frac{1}{M_{1\to1}} \right\vert^{2}$ | (13) |

In addition, obtaining the $T$ and $R$ spectrum of the structures enables the absorption ($A$) spectrum to be obtained by the following:

| $A=1-(T+R)$ | (14) |
| --- | --- |

In the structure given in Supplementary Figure 1, when the thickness of the conductive layers is zero, the structure is reduced to the same structure as PC systems, which consist of periodically arranged structures with different refractive indices. In this case, the Bragg wavelength $\lambda_{B}$, which corresponds to the resonance wavelength of the PBG formed by the PC, must meet the following condition:

| $\frac{\lambda_{B}}{4}=n_{i}d_{i}$ | (15) |
| --- | --- |

where $n_{i}$ is the real part of the refractive index of each layer and $d_{i}$ is the layer thickness.

**Average Visible Transmittance**

The transparency properties of DMD structure are determined by both AVT values and by transmittance characteristics in the visible light wavelength range (370–740 nm), taking into account the photonic response of the human eye ($V (\lambda))$, which is given in Supplementary Figure 2. The AVT value is calculated by the following formula^5,6^:

| $AVT=\frac{\int_{370 nm}^{780 nm} T\left( \lambda\right) V\left( \lambda\right) S_{AM1.5G}\left( \lambda\right) d\lambda}{\int_{370 nm}^{780 nm} V\left( \lambda\right) S_{AM1.5G}\left( \lambda\right) d\lambda}$ | (16) |
| --- | --- |

where $S_{AM1.5G}\left( \lambda\right)$ is the photon flux under AM 1.5G illumination, which is given in Supplementary Figure 2.


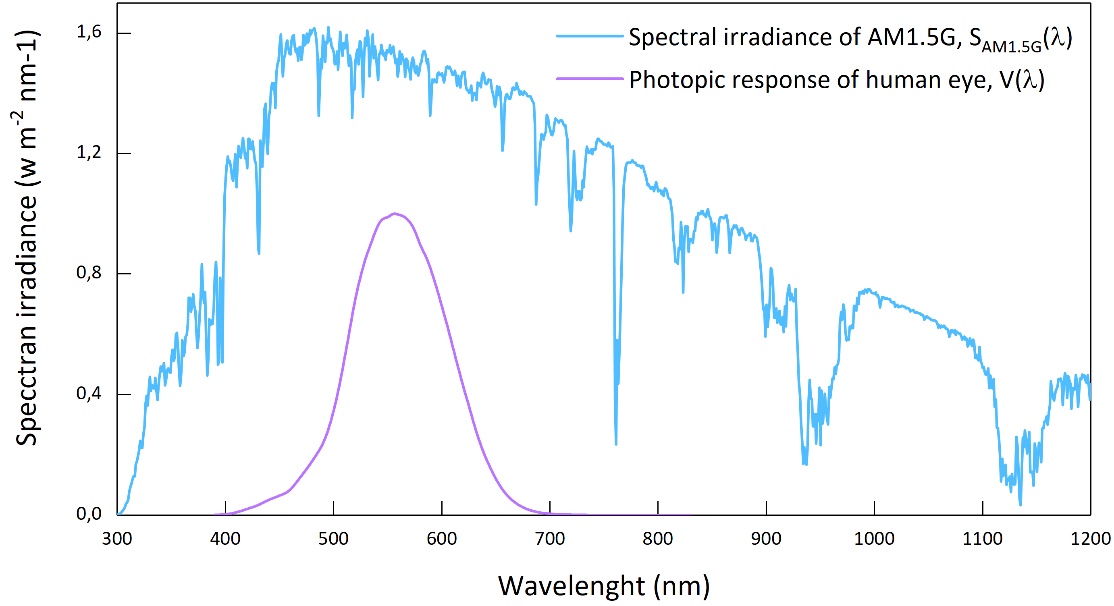


**Supplementary Figure 2.** Spectral distribution of photonic response of human eye and AM1.5G spectral irradiance.

AVT values depend on the working environment of transparent optoelectronic devices, and an AVT value of 25% is an acceptable criterion for window applications^5,7^. In addition, if an experimental result is to be obtained in AVT calculation, the $T(\lambda)$value to be used should be experimental, and the beam spot must be within the effective area during the measurement. If the spot area is larger than the effective area, some of the incident light may directly reach the detector and experimentally create an error in the transmittance measurement^5,8^.

**Calculation of CIE 1931 Color Coordinates**

In the CIE 1931 tristimulus system, $X$, $Y$, and $Z$ values can be calculated under VR with the following equations:

| $X=\int_{370 nm}^{780 nm} S_{AM1.5G}^{D65}\left( \lambda\right) T\left( \lambda\right) \bar{x}\left( \lambda\right) d\lambda$ | (17) |
| --- | --- |
| $Y=\int_{370 nm}^{780 nm} S_{AM1.5G}^{D65}\left( \lambda\right) T\left( \lambda\right) \bar{y}\left( \lambda\right) d\lambda$ | (18) |
| $Z=\int_{370 nm}^{780 nm} S_{AM1.5G}^{D65}\left( \lambda\right) T\left( \lambda\right) \bar{z}\left( \lambda\right) d\lambda$ | (19) |

In all these equations, $S_{AM1.5G}^{D65}$ is the CIE standard D65 illuminant spectrum, and the terms $\bar{x}\left( \lambda\right), \bar{y}\left( \lambda\right)$ and$\bar{z}\left( \lambda\right)$ are color-matching functions, which are given in Supplementary Figure 3, defined by the CIE protocol. $\left( X+Y+Z \right)=1$ and the color coordinates can be simplified to two-dimensional coordinates^9^:

| $x=\frac{X}{(X+Y+Z)}$ | (20) |
| --- | --- |
| $y=\frac{Y}{(X+Y+Z)}$ | (21) |


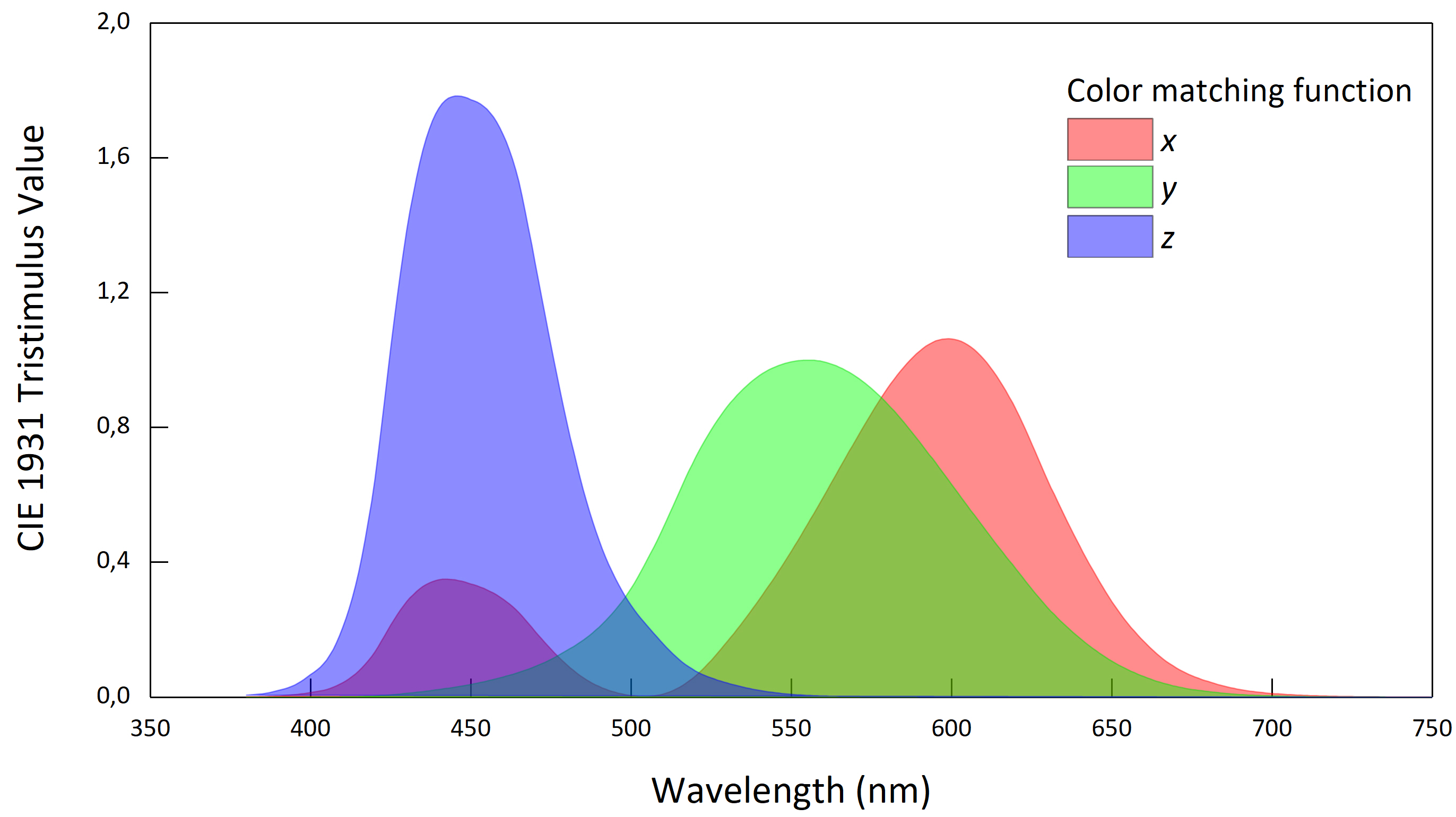


**Supplementary Figure 3.** Spectral distribution of $\bar{x}\left( \lambda\right), \bar{y}\left( \lambda\right)$ and$\bar{z}\left( \lambda\right)$ color-matching function.

**Color Rendering Index**

CRI values are calculated directly using the spectral distributions of the test lamp and reference illuminator^10^. The calculation is based on the spectral distributions of the test color sample (TCS) and a CRI value can be calculated for each test sample. The CRI of the test lamp can be calculated by averaging the calculated CRI values for each TCS^10^. The CRI values of the test source determined by averaging can be determined based on 8 TCS plus 7 additional TCS^10,11^.

For the evaluation of transparent structures in the CRI metric, the tristimulus values obtained from the transmittance spectrum of ST structures are used instead of the tristimulus values obtained from the reflectance spectrum of test lamps. Thus, a CRI assessment can be made for a construct with an ST.

To calculate the color difference, colorimetric values in the uniform color space are used in the CIE 1964 standards^10^:

|  | $W^{*}=25Y^{\frac{1}{3}}-17$  $U^{*}=13(u-u_{n})$  $V^{*}=13(v-v_{n})$ | (22) |
| --- | --- | --- |

where $u$ and $v$ are the color coordinates in the CIE 1960 chromaticity diagram. $u_{n}$ and $v_{n}$ are the CIE 1960 color coordinates of the illuminator. The $u$ and $v$ values can be calculated from the $X, Y$ and $Z$ tristimulus values or from the CIE 1960 color coordinates using the following equations:

|  | $u=\frac{4X}{X+15Y+3Z}=\frac{4x}{-2x+12y+3}$ | (23) |
| --- | --- | --- |
|  | $v=\frac{6Y}{X+15Y+3Z}=\frac{6y}{-2x+12y+3}$ | (24) |

The color coordinates of the source or ST construct to be tested do not match the color coordinates of the reference illuminator (the components of the test source and reference illuminator are labeled with indices i and k, respectively). This difference is corrected by the color adaptation process. Color adaptation is done by the following sets of equations^10^:

|  | $u_{k}^{'}=u_{r}$  $v_{k}^{'}=v_{r}$  $u_{k,i}^{'}=\frac{10.872+0.404c_{r}\frac{c_{k,i}}{c_{k}}-4d_{r}\frac{d_{k,i}}{d_{k}}}{16.518+1.481c_{r}\frac{c_{k,i}}{c_{k}}-d_{r}\frac{d_{k,i}}{d_{k}}}$  $v_{k,i}^{'}=\frac{5.520}{16.518+1.481c_{r}\frac{c_{k,i}}{c_{k}}-d_{r}\frac{d_{k,i}}{d_{k}}}$ | (25) |
| --- | --- | --- |

where $u_{k}^{'}$ and $v_{k}^{'}$ are the color CIE 1960 color coordinates of the test source after color adaptation, $u_{r}$ and $v_{r}$ reference illuminator, $u_{k,i}^{'}$ and $v_{k,i}^{'}$ are the color CIE 1960 color coordinates of the test colors after color adaptation. $c_{k}$ and $d_{k}$ are coefficients obtained from the chromaticity diagram and are calculated by the following equations ^10^:

|  | $c=\frac{4-u-10v}{v}$  $d=\frac{1.708v+0.404-1.481u}{v}$ | (26) |
| --- | --- | --- |

The coefficients $c$and $d$ can be indexed according to the color coordinates of both the test source and the reference illuminator. In addition, $W, U$ and $V$values in CIE 1964 standards are calculated for each test color and by determining the tristimulus values of the illuminating source and the test source. By modifying the equations with indexing, the chromaticity values for the CIE 1964 standards can be presented with the following sets of equations^10^:

|  | $W_{r,i}^{*}=25Y_{r,i}^{1/3}$-17  $U_{r,i}^{*}=13W_{r,i}^{*}(u_{r,i}-u_{r})$  $V_{r,i}^{*}=13W_{r,i}^{*}(v_{r,i}-v_{r})$  $W_{k,i}^{*}=25Y_{k,i}^{1/3}$-17  $U_{k,i}^{*}=13W_{k,i}^{*}(u_{k,i}-u_{k})$  $V_{k,i}^{*}=13W_{k,i}^{*}(v_{k,i}-v_{k})$ | (27) |
| --- | --- | --- |

According to the CIE 1964 uniform color space, Euclidean distance of each test color according to the illuminating source and the test source are calculated as follows^10^:

|  | $\Delta E_{i}=\sqrt{{(U_{r,i}^{*}-U_{k,i}^{*})}^{2}+{(V_{r,i}^{*}-V_{k,i}^{*})}^{2}+{(W_{r,i}^{*}-W_{k,i}^{*})}^{2}}$ | (28) |
| --- | --- | --- |

The color rendering indices for all test colors can be calculated by the Euclidean distance to the test colors^10^:

|  | $R_{i}=100-4.6\Delta E_{i}$ | (29) |
| --- | --- | --- |

The color rendering index of each test color can be used to derive an overall color rendering index^10^.

|  | $R_{g}=\frac{1}{N}\sum_{i=1}^{N} R_{i}$ | (30) |
| --- | --- | --- |

the index$i$ takes on different values up to the number ($N$) of the test colors of interest.

**Correlated Color Temperature**

The majority of light sources, including natural daylight, do not exhibit light color that is exactly on the blackbody curve. Because small deviations from the blackbody curve occur due to atmospheric absorption or production variability of artificial light sources. The correlational definition of color temperature describes a statistical process that these deviations reveal. This situation necessitated the calculation of correlated color temperature (CCT) closest to the black body instead of the necessity of determining the CCT on the black body curve. The deviation or proximity criterion is determined by the condition that $\Delta_{u,v}$<0.054 in CIE1960 color coordinates^10^. In the case of $\Delta_{u,v}$>0.054, i.e. where the deviations are high, mathematical association with a color temperature value is still possible. However, if the deviation is that great, the light source is no longer considered "white".

Using CIE1931 color coordinates, the CCT value can be calculated with McCamy approximation^12^ as follows:

|  | $CCT=-449{m\left( x,y \right)}^{3}+3525 {m\left( x,y \right)}^{2}+6823.3 m(x,y)+5520.33$ | (31) |
| --- | --- | --- |

where $m(x,y)=\left( \frac{x-0.3320}{y-0.1858} \right)$. As the CCT value increases, there is a shift down the blackbody curve and to the left, that is, towards the blue region.

**Supplementary Figure 4**


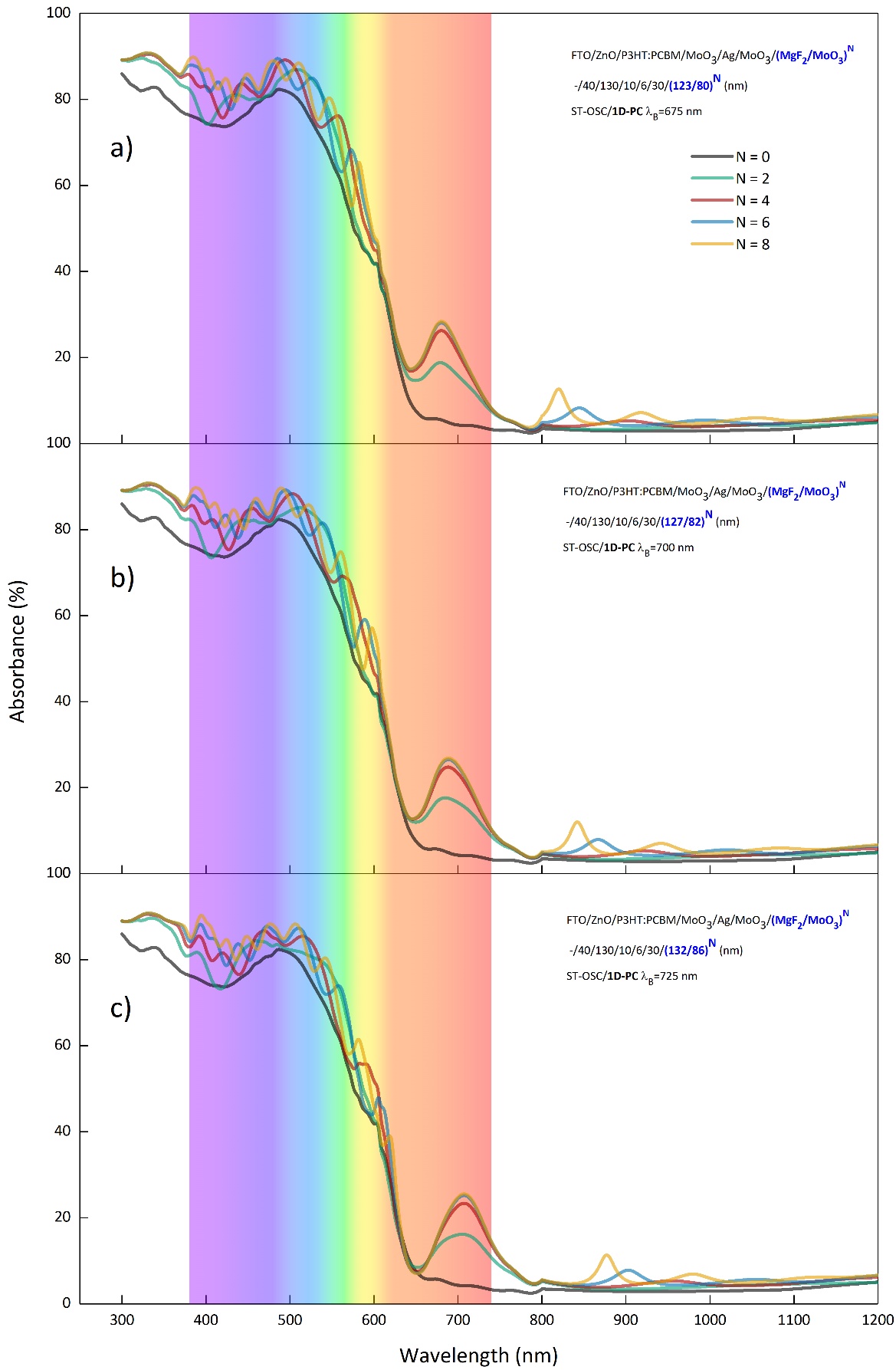


**Supplementary Figure 4.** Absorption spectra of (MgF_2_/MoO_3_)^N^ 1D-PC system with N=2, 4, 6 and 8 periods for **a)** $\lambda_{B}$=675nm, **b)** 700 nm and **c)** 725 nm.

**Supplementary Figure 5**

**
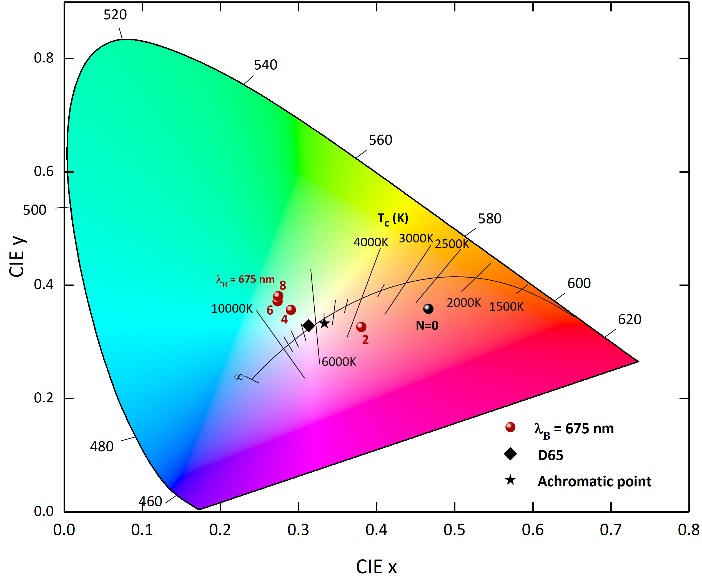
**

a)

b)


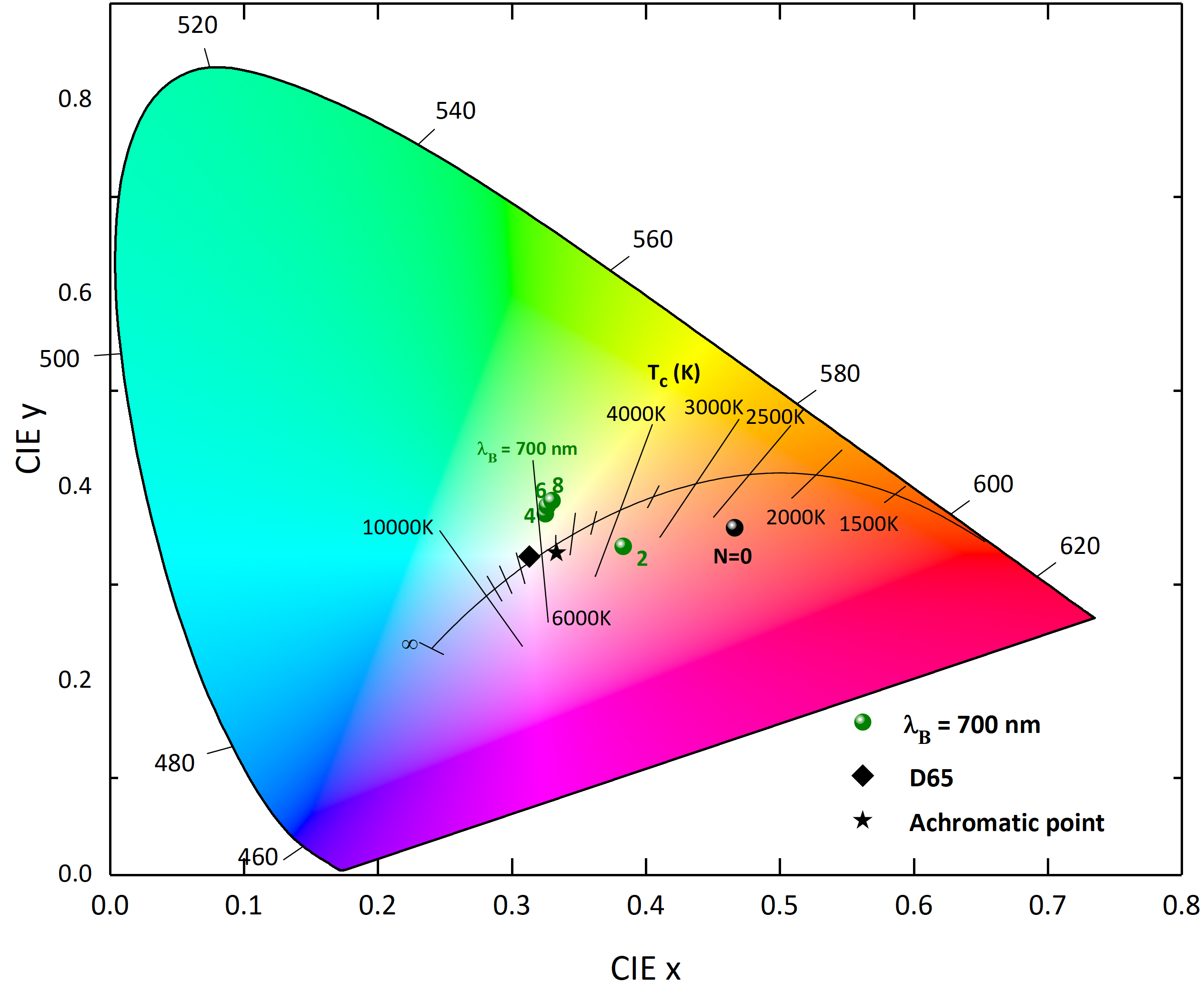


c)


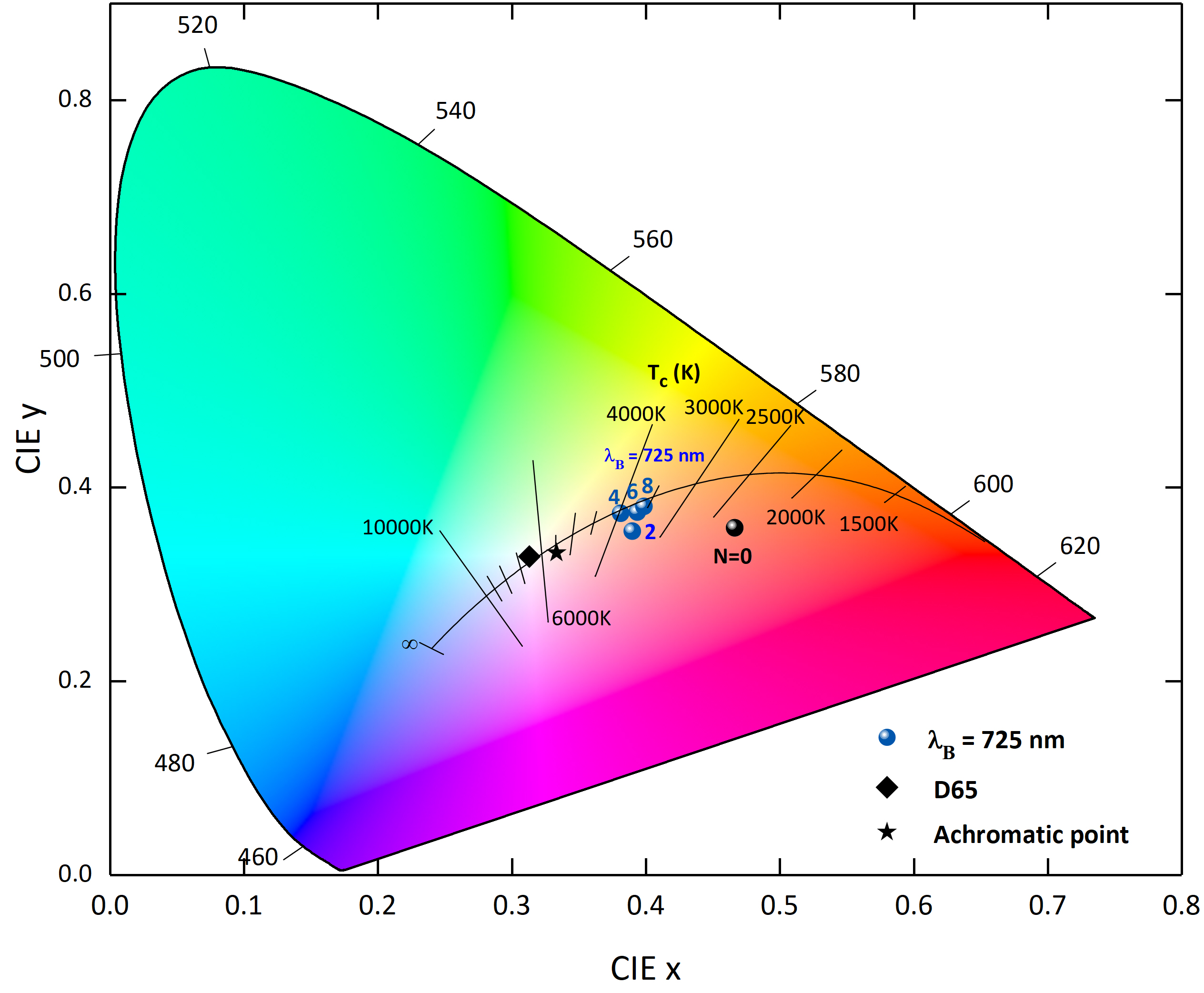


**Supplementary Figure 5.** Representation of ST-OSC/(MgF_2_/MoO_3_)^N^ in CIE 1931 chromaticity diagram of the variation of color coordinates with N values for **a)** $\lambda_{B}$==675 nm, **b)** 700 nm and **c)** 725 nm.

**Supplementary Figure 6**


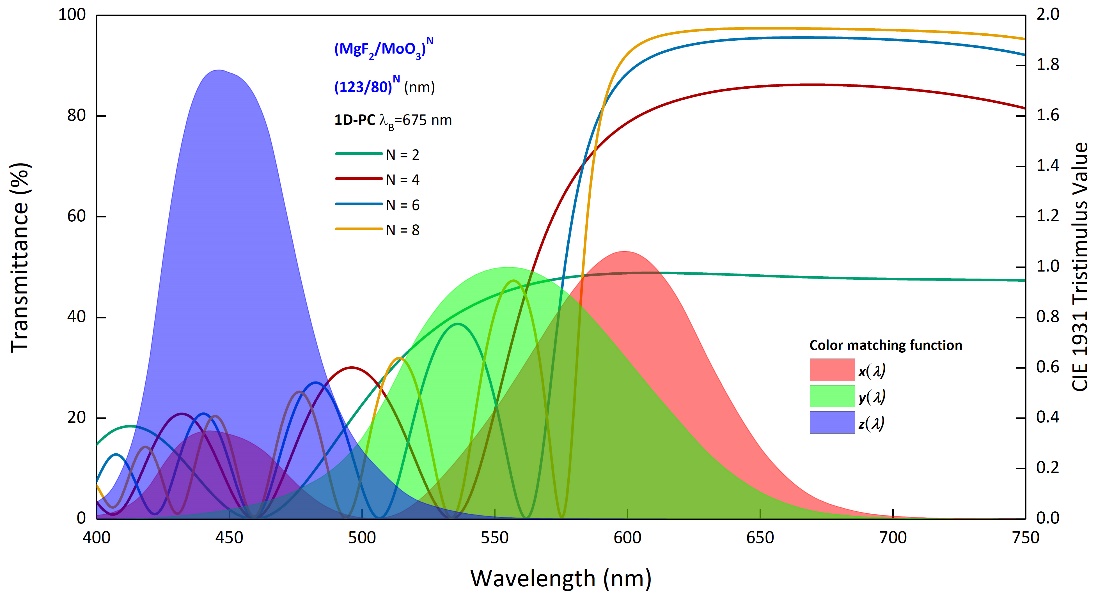


a)


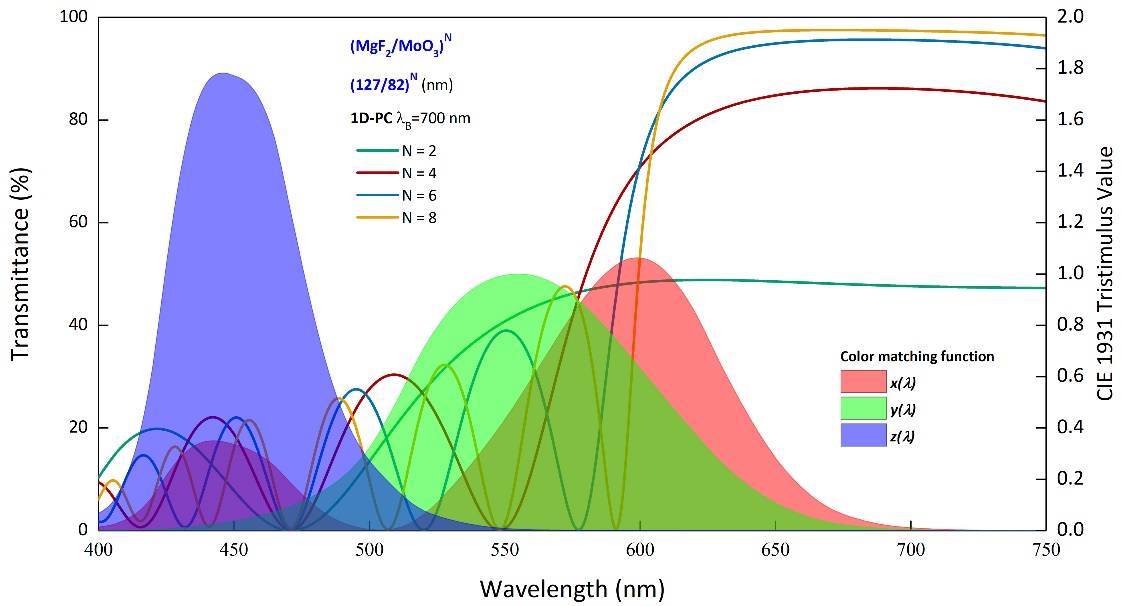


b)


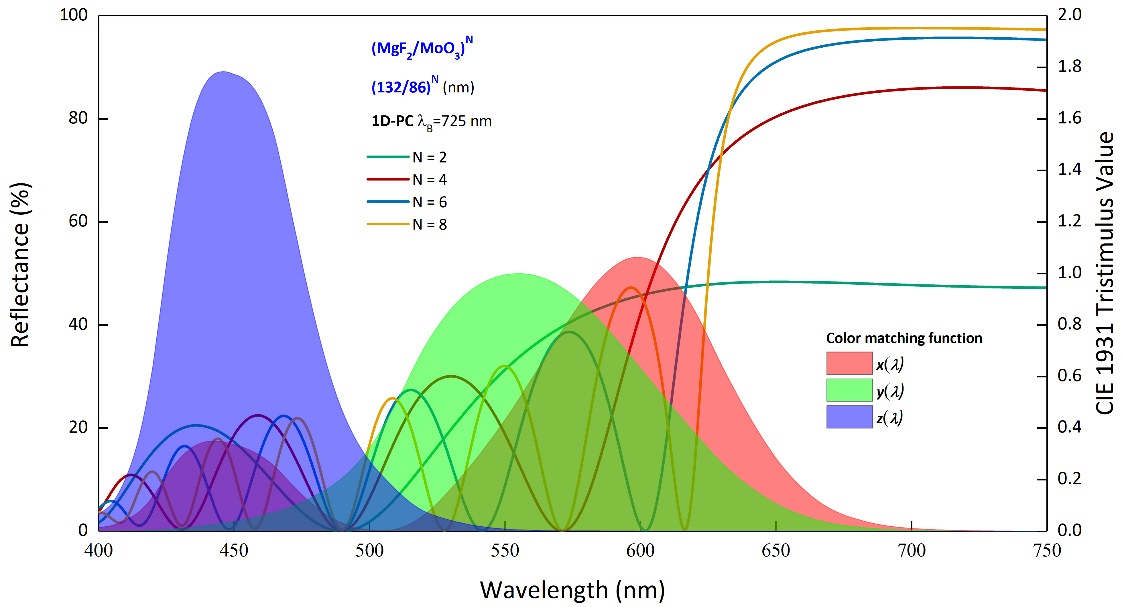


c)

**Supplementary Figure 6.** Transmittance spectra of the (MgF_2_/MoO_3_)^N^ 1D-PC structure **a)** $\lambda_{B}$=675 nm **b)** 700 nm and **c)** N=2 at 725 nm for periods 4, 6 and 8 in the range of 400-750 nm distribution of color matching functions along with

**Supplementary Figure 7**

a)


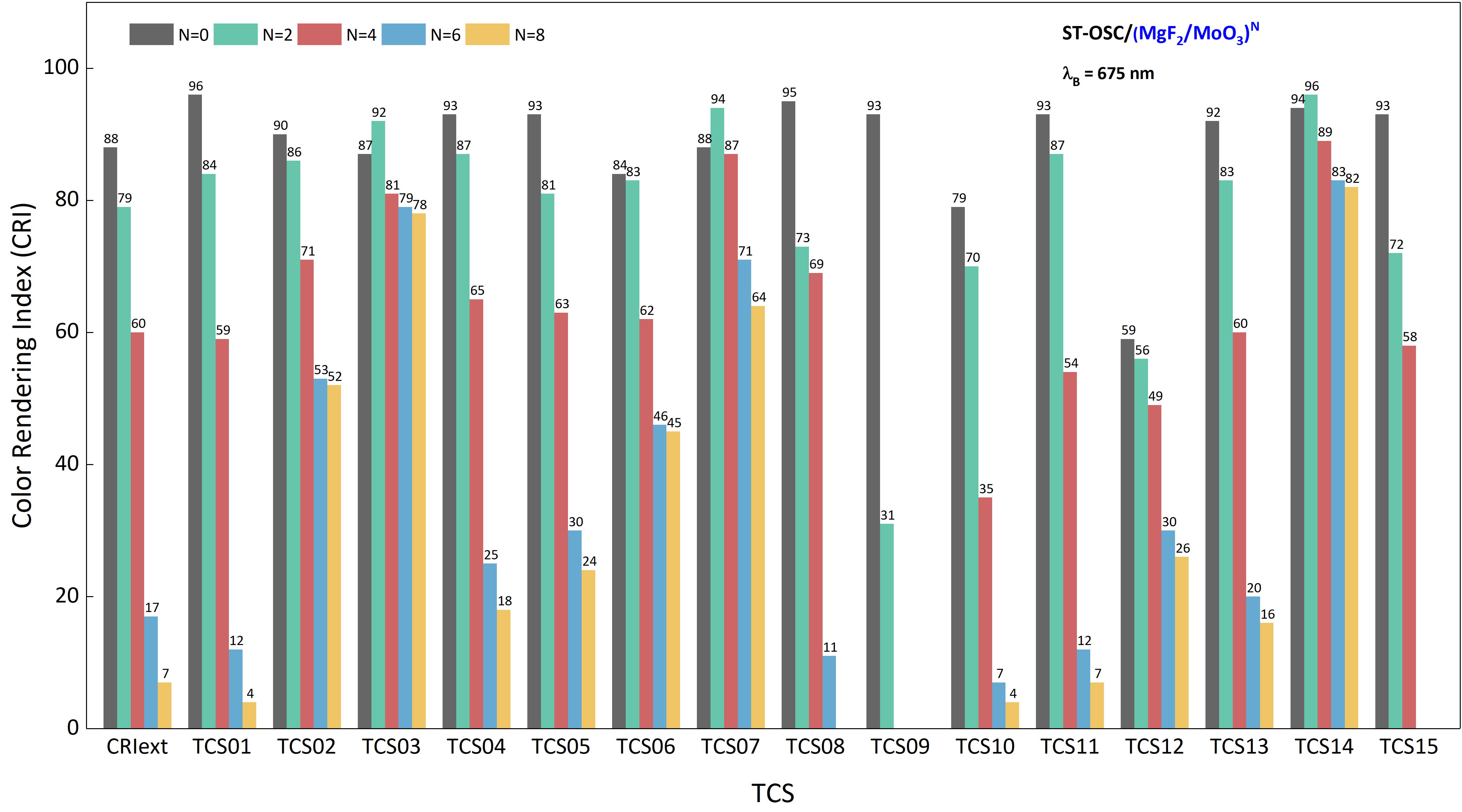


b)


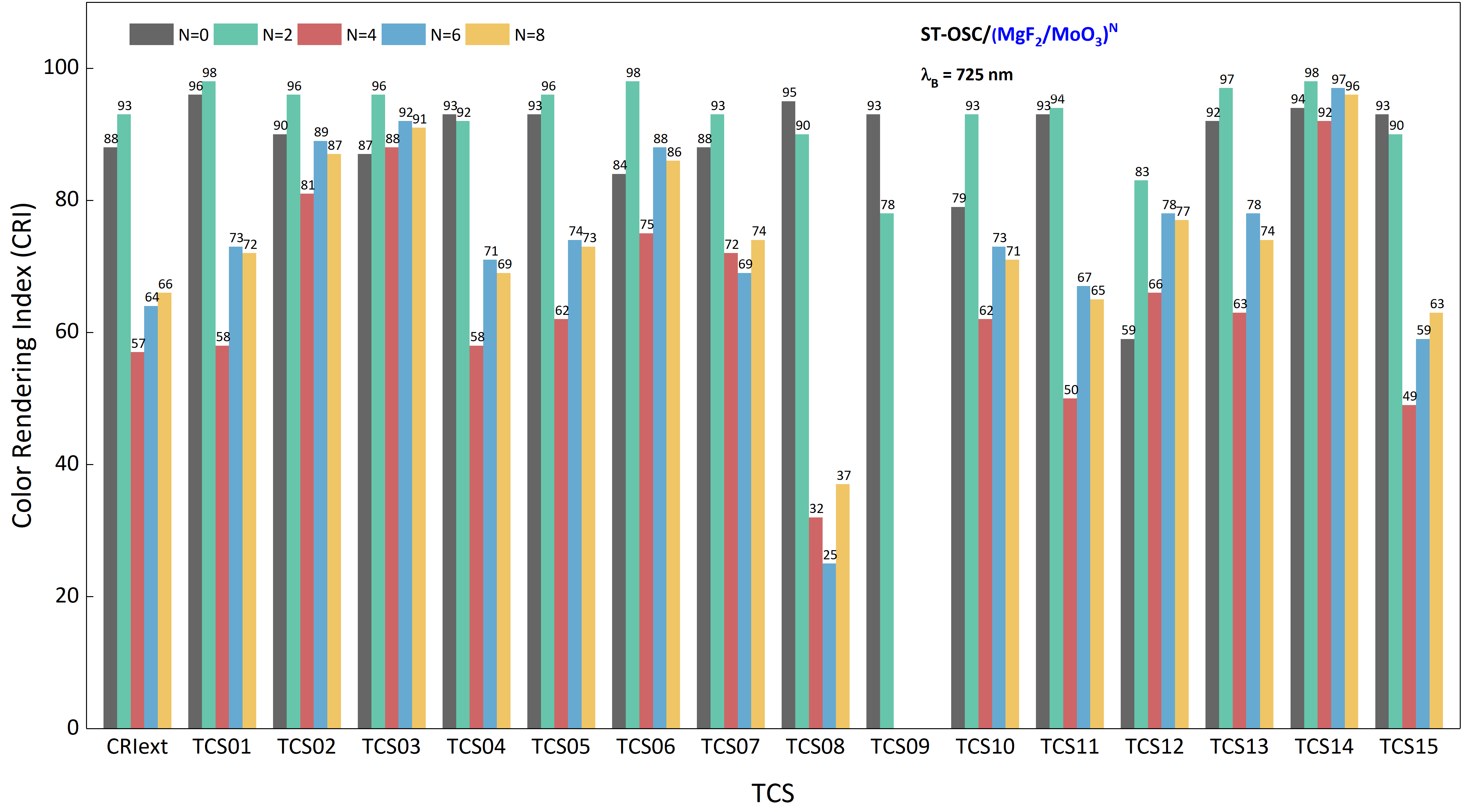


**Supplementary Figure 7.** CRI values for ST-OSC/(MgF_2_/MoO_3_)^N^ at N=2, 4, 6, 8 periods and **a)** $\lambda_{B}$==675 nm, **b)** 725 nm.

**Supplementary Figure 8**


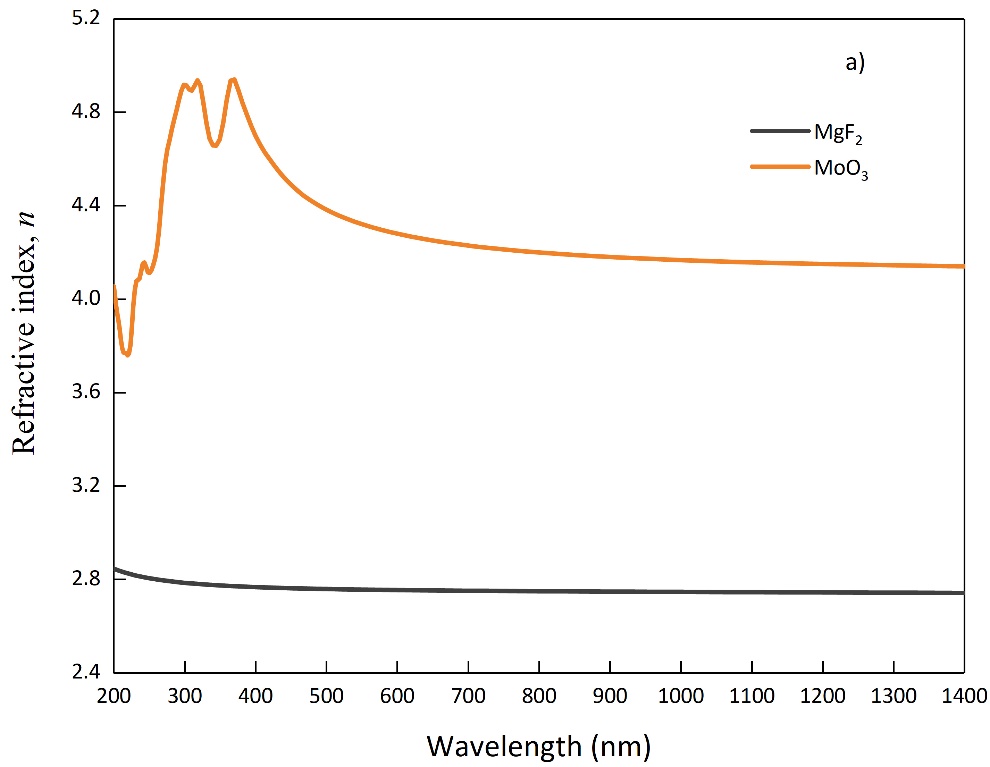


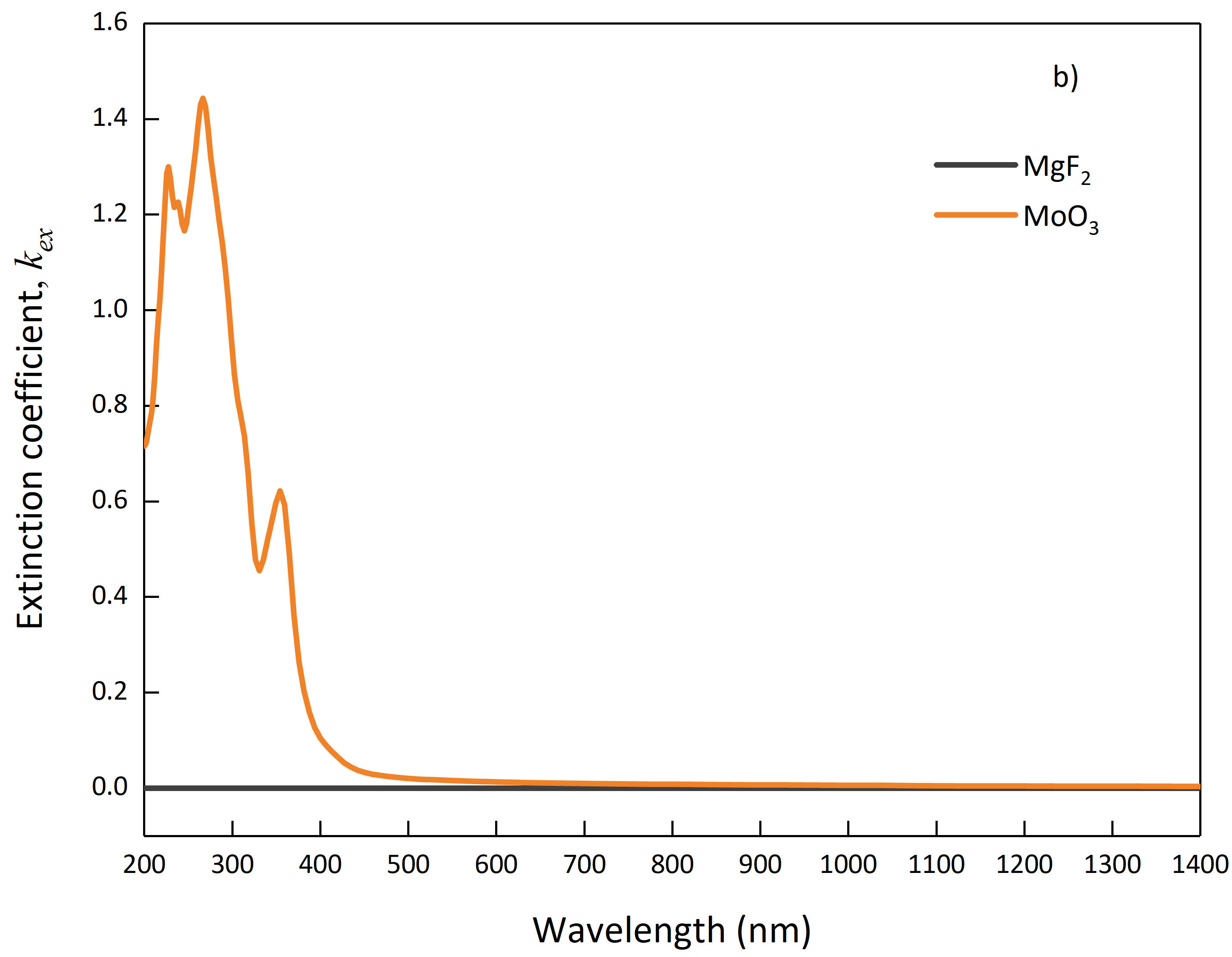


**Supplementary Figure 8.** Variations of **a)** refractive indices and **b)** extinction coefficients of MgF_2_ and MoO_3_ depending on wavelength.

**Supplementary Figure 9**

**
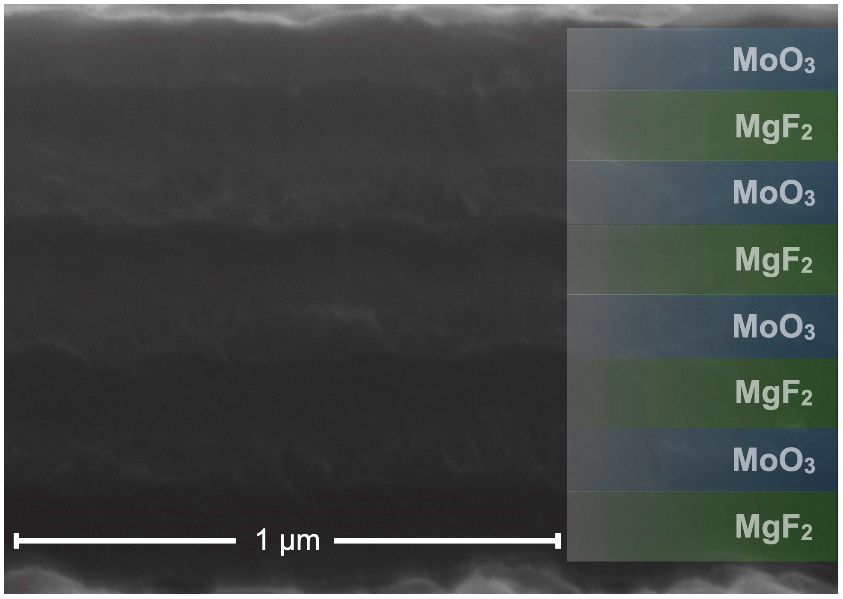
**

**Supplementary Figure 9.** Cross-section SEM image of (MgF_2_/MoO_3_)^4^ 1D-PC

**Supplementary Figure 10**


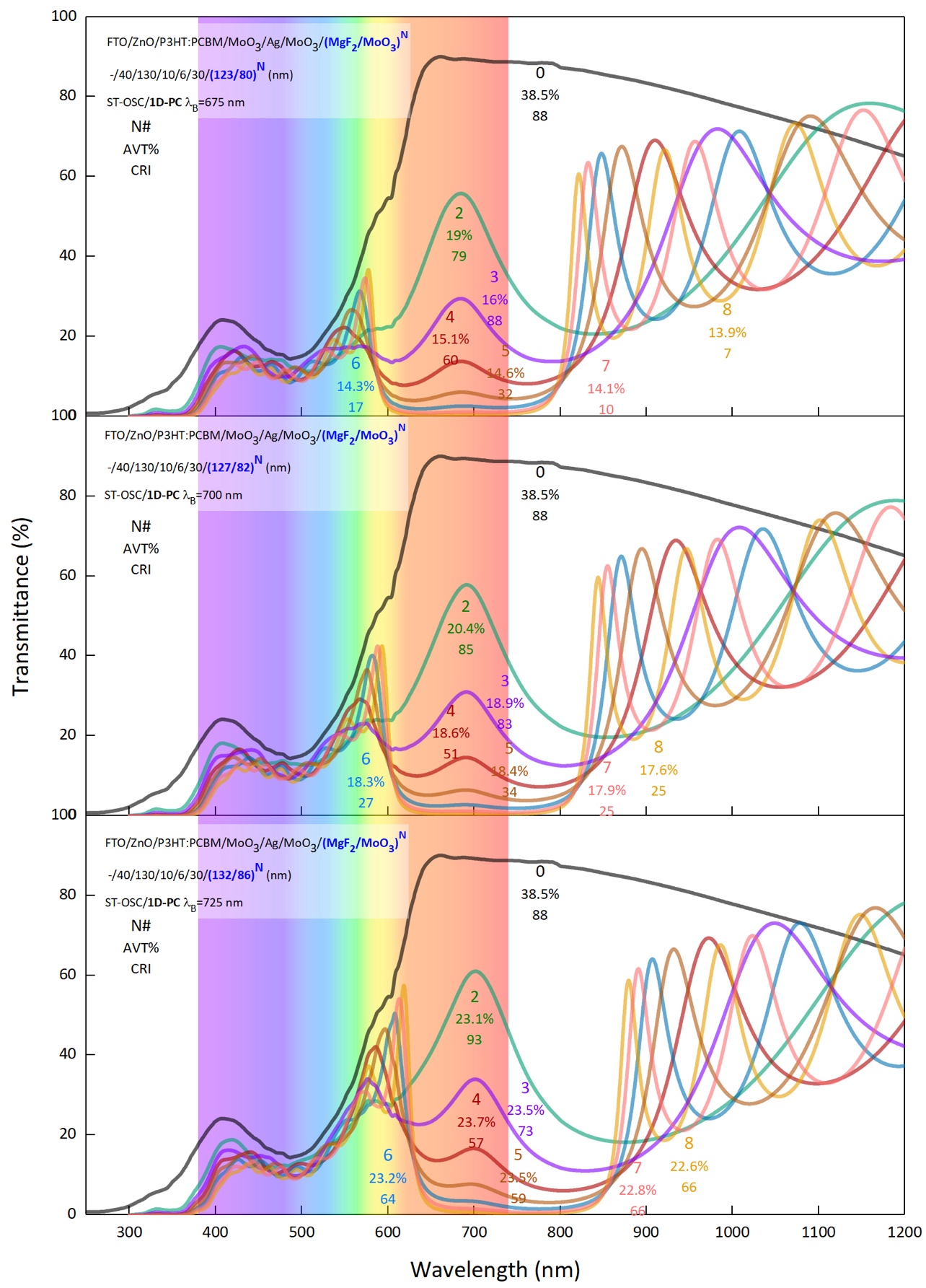


**Supplementary Figure 10.** Transmittance spectra of ST-OSC/(MgF_2_/MoO_3_)^N^ for N=2, 3, 4, 5, 6, 7, and 8 periods.

**Supplementary References**

1. Zhan, T., Shi, X., Dai, Y., Liu, X. & Zi, J. Transfer matrix method for optics in graphene layers. *Journal of Physics: Condensed Matter* **25**, 215301 (2013).

2. Yan, H. *et al.* Tunable infrared plasmonic devices using graphene/insulator stacks. *Nature Nanotechnology* **7**, 330–334 (2012).

3. Bonaccorso, F., Sun, Z., Hasan, T. & Ferrari, A. C. Graphene photonics and optoelectronics. *Nature Photonics* **4**, 611–622 (2010).

4. Yeh, P. & Hendry, M. Optical Waves in Layered Media. *Physics Today* **43**, 77–78 (1990).

5. Hu, Z. *et al.* A critical review on semitransparent organic solar cells. *Nano Energy* **78**, 105376 (2020).

6. Brus, V. v. *et al.* Solution‐Processed Semitransparent Organic Photovoltaics: From Molecular Design to Device Performance. *Advanced Materials* **31**, 1900904 (2019).

7. Chen, K.-S. *et al.* Semi-transparent polymer solar cells with 6% PCE, 25% average visible transmittance and a color rendering index close to 100 for power generating window applications. *Energy & Environmental Science* **5**, 9551 (2012).

8. Yang, C., Liu, D., Bates, M., Barr, M. C. & Lunt, R. R. How to Accurately Report Transparent Solar Cells. *Joule* **3**, 1803–1809 (2019).

9. Shin, D. & Choi, S.-H. Recent Studies of Semitransparent Solar Cells. *Coatings* **8**, 329 (2018).

10. Ohta, N. & Robertson, A. R. *Colorimetry: Fundamentals and Applications*. (John Wiley & Sons, 2005).

11. Li, Z., Ma, T., Yang, H., Lu, L. & Wang, R. Transparent and Colored Solar Photovoltaics for Building Integration. *Solar RRL* **5**, 1–21 (2021).

12. Kelly, K. L. Lines of Constant Correlated Color Temperature Based on MacAdam’s (u,υ) Uniform Chromaticity Transformation of the CIE Diagram. *J Opt Soc Am* **53**, 999 (1963).
